# Combined exome and transcriptome sequencing of non-muscle-invasive bladder cancer: associations between genomic changes, expression subtypes, and clinical outcomes

**DOI:** 10.1186/s13073-022-01056-4

**Published:** 2022-06-03

**Authors:** Anshita Goel, Douglas G. Ward, Boris Noyvert, Minghao Yu, Naheema S. Gordon, Ben Abbotts, John K. Colbourne, Stephen Kissane, Nicholas D. James, Maurice P. Zeegers, Kar Keung Cheng, Jean-Baptiste Cazier, Celina M. Whalley, Andrew D. Beggs, Claire Palles, Roland Arnold, Richard T. Bryan

**Affiliations:** 1 Bladder Cancer Research Centre, University of Birmingham, Birmingham, UK; 2 Institute of Cancer and Genomic Sciences, University of Birmingham, Birmingham, UK; 3 Centre for Computational Biology, University of Birmingham, Birmingham, UK; 4 CRUK Birmingham Centre, University of Birmingham, Birmingham, UK; 5 School of Biosciences, University of Birmingham, Birmingham, UK; 6 Institute of Cancer Research, London, UK; 7 The Royal Marsden NHS Foundation Trust, London, UK; 8 Department of Complex Genetics and Epidemiology, School of Nutrition and Translational Research in Metabolism, Maastricht University, Maastricht, The Netherlands; 9 CAPHRI School for Public Health and Primary Care, University of Maastricht, Maastricht, The Netherlands; 10 Institute of Applied Health Research, University of Birmingham, Birmingham, UK; 11 Genomics Birmingham, University of Birmingham, Birmingham, UK

**Keywords:** Urothelial carcinoma, Bladder cancer, Exome, Transcriptome, Sequencing, Mutations, Subtypes, Prognosis, Therapy

## Abstract

**Background:**

Three-quarters of bladder cancer patients present with early-stage disease (non-muscle-invasive bladder cancer, NMIBC, UICC TNM stages Ta, T1 and Tis); however, most next-generation sequencing studies to date have concentrated on later-stage disease (muscle-invasive BC, stages T2+). We used exome and transcriptome sequencing to comprehensively characterise NMIBCs of all grades and stages to identify prognostic genes and pathways that could facilitate treatment decisions. Tumour grading is based upon microscopy and cellular appearances (grade 1 BCs are less aggressive, and grade 3 BCs are most aggressive), and we chose to also focus on the most clinically complex NMIBC subgroup, those patients with grade 3 pathological stage T1 (G3 pT1) disease.

**Methods:**

Whole-exome and RNA sequencing were performed in total on 96 primary NMIBCs including 22 G1 pTa, 14 G3 pTa and 53 G3 pT1s, with both exome and RNA sequencing data generated from 75 of these individual samples. Associations between genomic alterations, expression profiles and progression-free survival (PFS) were investigated.

**Results:**

NMIBCs clustered into 3 expression subtypes with different somatic alteration characteristics. Amplifications of *ARNT* and *ERBB2* were significant indicators of worse PFS across all NMIBCs. High APOBEC mutagenesis and high tumour mutation burden were both potential indicators of better PFS in G3pT1 NMIBCs. The expression of individual genes was not prognostic in BCG-treated G3pT1 NMIBCs; however, downregulated interferon-alpha and gamma response pathways were significantly associated with worse PFS (adjusted *p*-value < 0.005).

**Conclusions:**

Multi-omic data may facilitate better prognostication and selection of therapeutic interventions in patients with G3pT1 NMIBC. These findings demonstrate the potential for improving the management of high-risk NMIBC patients and warrant further prospective validation.

**Supplementary Information:**

The online version contains supplementary material available at 10.1186/s13073-022-01056-4.

## Background

Urothelial bladder cancer (UBC) is a complex disease both clinically and at the molecular level. Muscle-invasive bladder cancer (MIBC, UICC TNM stages T2+ [1]) is associated with a poor prognosis which becomes progressively worse with disease extent—these tumours are aggressive, and their genomic and epigenomic aberrations have been extensively studied in recent years [2]. Although some genomic events occur with high frequency (e.g. *TERT* promoter mutations in > 75% of cases, *TP53* mutations in c.50%), MIBCs are heterogeneous and characterised by a large number of single nucleotide variants (SNVs) and copy number variants (CNVs) [2, 3]; loss of multiple tumour suppressors and alteration of multiple pathways are common. Gene expression profiling studies have classified MIBCs into six consensus subtypes which share some characteristics [4], but which remain heterogeneous with respect to genomic aberrations and behaviour; temporal and spatial plasticity in subtype has also been reported [5].

Non-muscle-invasive bladder cancer (NMIBC, stages Ta/T1/Tis [1]) accounts for over 75% of UBCs at presentation and is arguably more complex than MIBC, comprising multiple grades of disease (based upon cellular appearances determined by microscopy) from well-differentiated (grade 1, G1) to poorly differentiated (grade 3, G3) [6]. Whilst the vast majority of MIBCs are G3 or high grade, NMIBCs range from lower grade Ta tumours (the majority of which are indolent) to higher grade T1 tumours (with considerable risk of progression to both muscle invasion and metastasis) [7]. Given our current understanding, it is not yet possible to accurately predict which of the higher grade NMIBCs will progress to the invasive form of the disease (MIBC) or lead to adverse outcomes (including cancer-related death).

Low-grade tumours ordinarily harbour relatively few genomic aberrations (often activating point mutations in oncogenes such as *FGFR3*, *PIK3CA* and *RAS* [8]), typically exhibit luminal expression subtypes, and can be subdivided into two groups on the basis of CNVs (GS1 and GS2) [9]. At the genomic level, high-grade NMIBCs are akin to MIBCs with many CNVs and SNVs, loss of tumour suppressor genes [10], and a range of basal or luminal expression profiles [11]. The accumulated evidence suggests that UBCs develop along two distinct pathways—papillary low-grade tumours developing from intermediate cells and non-papillary high-grade tumours developing from basal urothelial cells [12, 13]; notwithstanding, early events appear common to both pathways (e.g. *TERT* promotor mutations, loss of *CDKN2A*) [14, 15]. Additionally, some low-grade tumours may acquire additional genomic aberrations which transform them into aggressive high-grade tumours [16].

Compared to MIBC, next-generation sequencing studies of NMIBC have been much more limited; amongst the largest exome or whole-genome sequencing studies are Guo et al. [17], Nordentoft et al. [18], Wu et al. [19] and Hurst et al. [9] (37, 20, 21 and 24 cases, respectively). Some studies have investigated larger NMIBC cohorts using targeted cancer gene panels [20, 21], and Lindskrog et al. used RNA sequencing (RNA-seq) data to cluster 535 NMIBCs into four gene expression classes with differing clinical outcomes [22]. Regarding prognosis in high-risk NMIBC, Bellmunt et al. exome sequenced 62 high-grade T1 tumours and analysed mutations in 95 bladder-cancer associated genes [23], Robertson et al. proposed a 5-class prognostic classifier based on RNA-seq of 73 G3pT1s subsequently treated with BCG [24] and Damrauer et al. [25] have identified a tumour microenvironment expression signature that shows promise for predicting response to BCG.

In the current study, we have performed the largest overlapping exome and RNA sequencing of NMIBC to date. The patient cohort comprised 96 prospectively collected fresh-frozen NMIBCs spanning a range of stages and grades with a median of 4.96 years’ clinical follow-up [26]. We report on SNVs, indels, CNVs, mutation signatures, gene expression subtypes and their impact on clinical outcomes. We identify several prognostic genomic factors that warrant further validation in NMIBC generally and in G3pT1 disease specifically.

## Methods

### Patients and samples

Patients were recruited consecutively from 2005 to 2010 from ten hospitals in the West Midlands (UK) as part of the Bladder Cancer Prognosis Programme (BCPP) [26]. Participants gave written informed consent for enrolment into the present study based upon initial cystoscopic findings suggestive of primary BC (UK national research ethics approval 06/MRE04/65, East Midlands - Derby Research Ethics Committee). The research was undertaken in accordance with the ethical standards of the 1964 Helsinki Declaration and its later amendments. All patients were newly diagnosed primary NMIBC cases, treatment-naïve at biospecimen collection and subsequently treated according to contemporary European Association of Urology (EAU) guidelines (including re-section where indicated) and EAU NMIBC risk group stratification [27–29]. Where necessary, tumour grade and stage records were amended according to the results of early re-resection or cystectomy. We used the 1973 grade classification as it was in universal use in the UK at the time of patient recruitment and is the basis for the EORTC and EAU NMIBC risk categories and has comparable utility to the 2004/2016 classification [6]. Tissues were collected at the time of transurethral resection of bladder tumour (TURBT), snap-frozen in liquid nitrogen in the operating theatre, and subsequently stored at −80 °C. All included tumours were purely or predominantly urothelial carcinomas and were classified according to grade (WHO 1973 [30]) and stage (UICC). During a median of 4.96 years’ clinical follow-up, 32 of the 96 patients died, with UBC recorded as the cause of death in 17 instances. Additionally, recurrence occurred in 50 cases and progression to MIBC in 26 cases. Patient demographics are shown in Additional file 1: Table S1. Tissues and blood were stored at −80 °C; DNA was extracted from 25 mg tissue and 100 μl paired blood using DNeasy Blood and Tissue kits and RNA from 25 mg frozen tissue using RNeasy kits (Qiagen, Hilden, Germany).

### Library preparation and sequencing

Of the 96 tumour samples, 93 yielded enough DNA for whole-exome sequencing (WES) with paired blood germline DNA and 78 enough high-quality RNA for RNA sequencing (RNA-seq) [31, 32]. Thus, 75 patients had overlapping data from both WES and RNA-seq; 18 patients had WES only, and 3 patients had RNA-seq only (75 + 18 + 3 = 96). Sequencing libraries were prepared using the Nextera® Rapid Capture Exome and TruSeq® Stranded RNA LT kits (Illumina, San Diego, USA) and HiSeq/NextSeq sequenced. The *TERT* promoter and the 5′ end of exon 7 of *FGFR3* were sequenced separately using PCR-based library preparation [33].

### Whole-exome sequencing (WES) data analysis

Reads were mapped to the human genome (GRCh37) using BWA [34]. BAM files were created using Picard tools and subjected to local realignment (using InDels and SNPs from 1000 Genomes) and base quality score recalibration using GATK [35]. Furthermore, a panel of normals (PoN) was created using the germline samples, and Mutect (v2.2) [36] was run for each tumour-normal pair using the PoN as well as polymorphic loci information from gnomAD [37]. VCFs were filtered using FilterMutectCalls (GATK) and annotated with Variant Effect Predictor [38]. The contributions of COSMIC mutational signatures [39] to the total SNV burden in each NMIBC were calculated using deconstructSigs [40]. Copy number changes were detected using CNVkit [41] on paired tumour-normal BAMs, and resultant copy number segments were processed using GISTIC [42] to identify focal copy number peaks.

Tumour cellularity and overall ploidy were estimated by Sequenza [43] from the paired tumour-normal BAM files. The Sequenza output was further analysed using the scarHRD package [44] to extract three indices of homologous recombination deficiency (HRD): telomeric allelic imbalance (HRD-TAI), loss-of-heterozygosity profiles (HRD-LOH) and large-scale state transitions (HRD-LST). The combined score (HRD score) from the sum of the three indices was used for downstream analyses. Full details of all software versions, settings and filtering steps are provided in Additional file 3: Additional Methods.

### RNA sequencing (RNA-seq) data analysis

Reads were mapped to the human reference genome (GRCh37) and transcriptome (annotation reference Ensembl release 87) using the STAR aligner [45]. Expression data were normalised using the voom method [46], and differential expression analysis was performed in the limma package in R. We carried out gene set enrichment analyses using the GAGE R Bioconductor package [47] based on MSigDB hallmark gene sets. The ConsensusClusterPlus R package [48] was used to stratify the RNA-seq samples into stable clusters. Details of the iterative process are provided in the Additional file 3: Additional Methods. Additionally, activities of 23 previously published regulons were assessed in the RNA-seq cohort using the RTN (Reconstruction of Transcriptional regulatory Networks and analysis of regulons) package [49]. Multivariate survival analyses utilised the Kaplan-Meier method implemented in R “survival” package. All statistical analyses and data visualization were carried out using R/Biocoductor packages. Comparisons of proportions between the groups were performed using Fisher’s exact tests; Mann-Whitney tests were used to compare the means between two groups and Kruskal-Wallis tests for more than two groups. Benjamini-Hochberg correction was used to control the false discovery rate for multiple testing. Further details on somatic mutation and copy number calling, mutational signature analysis, in silico circular RNA detection and prediction of immune cell infiltration status are provided in Additional file 3: Additional Methods.

## Results

### Exome analysis

#### Somatic mutations and copy number alterations

**Fig. 1:**
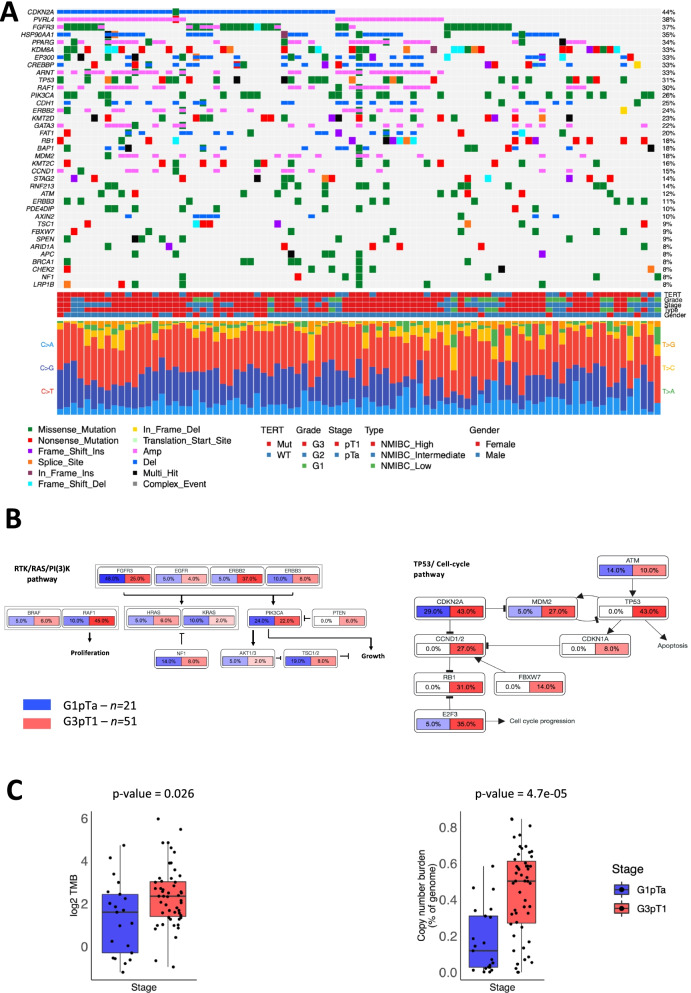
Recurrent genomic alterations and comparison of G1pTa and G3pT1 tumours. **A** The distribution of non-synonymous mutations and focal CNAs in COSMIC tier 1 genes found in ≥ 8 patients out of 93 NMIBC cases. The top panel of the figure shows the recurrently altered gene symbol on the left, the recurrence frequency on the right, and the area between them is divided into 93 columns, each representative of a NMIBC patient from the BCPP cohort. The individual colour codes within the cells are representative of the genomic alteration type. The middle panel of the figure represents *TERT* promoter mutation status (from targeted sequencing), tumour grade, tumour stage, NMIBC risk group, and gender. The third panel of the figure represents the six classes of base substitution (C>A, C>G, C>T, T>A, T>C, and T>G) frequency in each of the patients. **B** Comparative difference in alteration incidence (mutation and/or copy number alterations) amongst the RTK/RAS/PI(3)K and the TP53/cell cycle cellular pathways. Each constituent gene has two boxes beneath it in blue (left side) and red (right side) colours for G1pTa (*n* = 21) and G3pT1 (*n* = 51) tumours, respectively. The number within each of the boxes indicates the percentage of the patients altered (mutated/CNA) either in G1pTa (blue box) or G3pT1 (red box). If no alterations were detected, then the relevant boxes have no colour. **C** Boxplots comparing the overall distribution of tumour mutational burden (TMB; log2 transformed) and copy number alteration (CNA) burden in G1pTa (*n* = 21; blue colour) and G3pT1 (*n* = 51; red colour) samples. The *X*-axis is the stage (G1pTa or G3pT1), and the *Y*-axis is the aberration index: either TMB or CNB. Mann-Whitney tests were performed to assess the statistical significance, and the *p*-values are noted above each pair of boxplots

We obtained average exome read depths of 80× and 25× for tumour and germline DNA, respectively. Mutect2 identified a total of 31,086 somatic mutations (30,191 SNVs and 895 indels) which, after gene annotation and filtering for polymorphic loci, left 16,090 non-synonymous mutations (52%, 15,480 SNVs and 610 indels, Additional file 1: Table S2). Recurrent somatic alterations are summarised in Fig. 1A, Additional file 2: Fig. S1 and Additional file 1: Tables S2-S5. SNVs in the *TERT* promoter were the most prevalent alterations (77%) [33], followed by *CDKN2A* deletion (44%), *PVRL4* amplification (38%), *HSP90AA1* deletion (35%) and *PPARG* amplification (34%); *FGFR3* harboured SNVs in 33% of NMIBCs and amplifications in 9%. Chromatin modification genes were frequently mutated, including *EP300* (33%), *KDM6A* (33%), *KMT2D*/*C* (23%/16%) and *ARID1A* (8%). Loss-of-function *RB1* mutations (18%) and *CDKN2A* deletions (43%) indicate cell cycle checkpoint deficiency in at least 57% of NMIBCs.

#### Frequently altered pathways in G3pT1 tumours

Signalling pathways with multiple genes altered included RTK/RAS/PI3K (86% of tumours), histone modification (84%), TP53/cell cycle (77%), DNA damage repair (28%) and the cohesin complex (21%). *TP53*, *RB1*, *CCND1*/2, *CDKN1A* and *FBXW7* were exclusively altered in G3pT1 cases when compared to G1pTa; *E2F3* was amplified in 5% of G1pTa and 35% of G3pT1 NMIBCs (Fig. 1B). *ERBB2* and *RAF1* were altered in 37% and 45% of G3pT1 tumours, respectively, but only in 5% and 10% of G1pTa tumours, respectively. Conversely, *FGFR3* was more frequently altered in G1pTa NMIBCs (48% versus 25% for G3pT1; Fisher’s exact *p*-value = 0.095).

Tumour mutational burden (TMB) varied across grades and stages: G3pT1 cases had median TMB of 2.81 mutations/Mb versus 1.14 mutations/Mb for G1pTa (Mann-Whitney *p* = 0.026) (Fig. 1C). Copy number burden (CNB, percentage of the genome altered by CN segments) ranged from 0.02 to 84.6% (median 32.5%) and was higher in G3pT1 disease (median 50.3%) than in G1pTa (median 11.9%) (Mann-Whitney *p* = 4.7e−05) (Fig. 1C).

#### APOBEC mutational signatures are abundant in NMIBCs

**Fig. 2:**
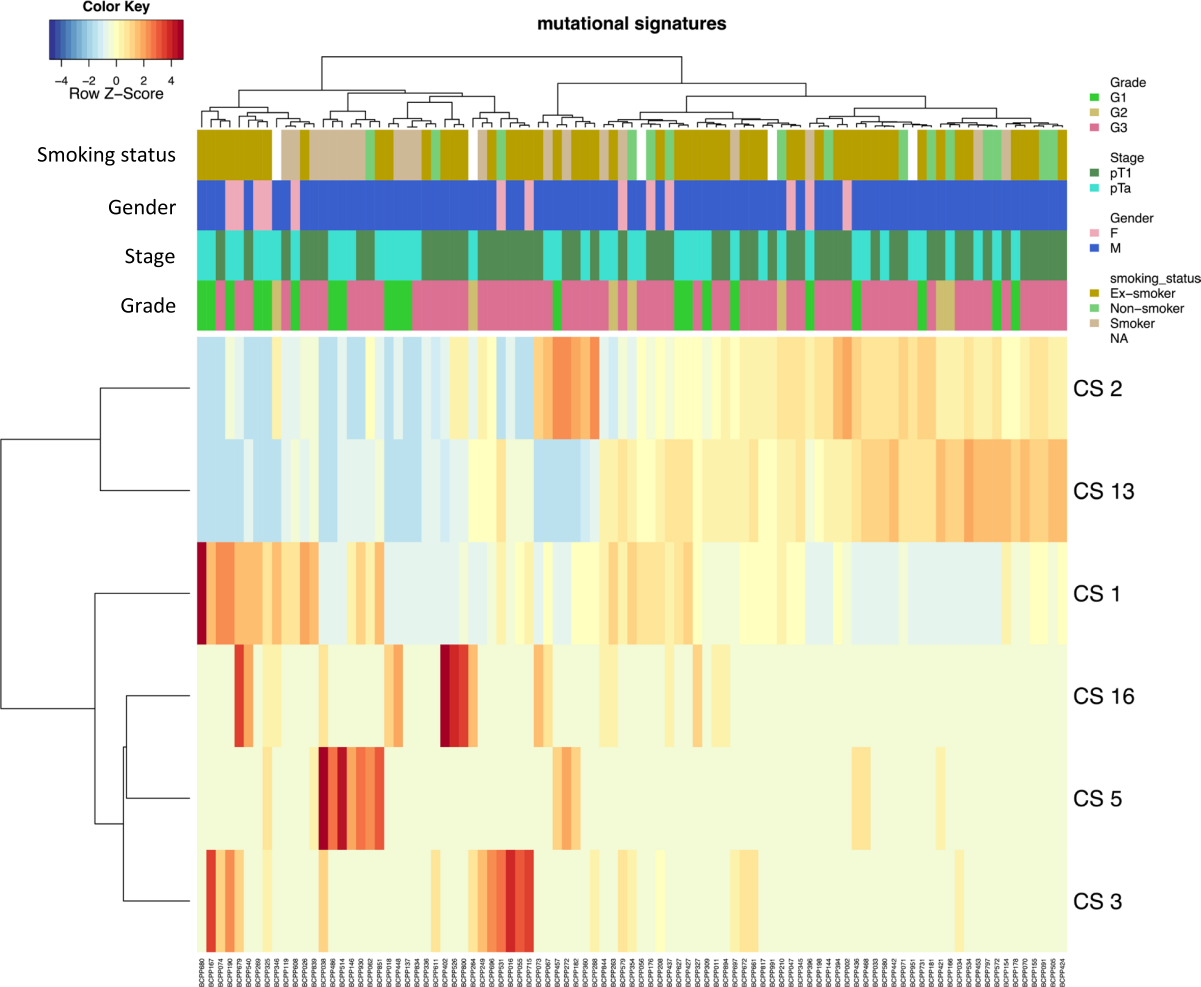
Single base substitution-based mutational signatures prevalent in BCPP NMIB cohort. The *x*-axis represents the 93 NMIBC samples, and the *y*-axis (right side) denotes the six COSMIC mutational signatures (ver.2) with ≥ 10% contribution in at least 10 samples. Additionally, above the heatmap are annotation ribbons for clinical phenotype features of tumour grade, stage, gender, and smoking status

Estimation of relative contribution of COSMIC SBS (single base substitution) signatures (CS) per sample revealed APOBEC-related CS 2 and 13 together 111 to have likely contributed 48% of mutations on average (0% in 10 NMIBCs, > 80% in 13 NMIBCs). Other signatures present with > 10% contribution in ≥ 10 samples included CS 1 (12% of NMIBCs), CS 3 (4%), and CS 5 and CS 16 (both at 3%). The cohort clustered into two groups (Fig. 2): APOBEC-low (high CS 1 and/or 3, 5 or 16) (*n* = 36) and APOBEC-high (high CS 2 and/or 13) (*n* = 57). No associations (as per Fisher’s exact test) were observed between these groups and tumour stage (*p* = 0.672), grade (*p* = 0.349), smoking status (*p* = 0.085) or gender (*p* = 0.239).

#### Exome sequencing identifies prognostic features in NMIBC

**Fig. 3:**
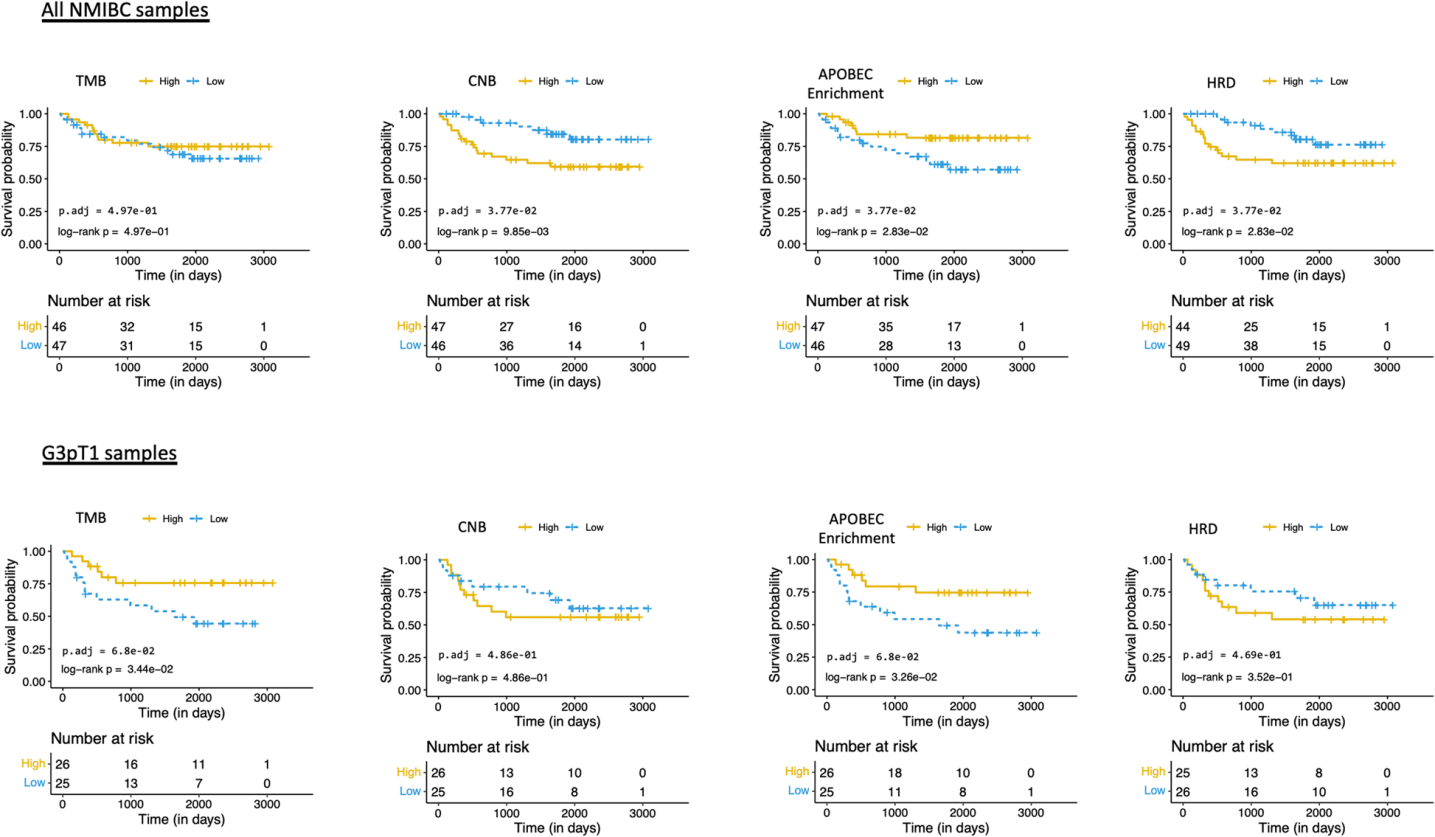
Univariate survival analysis. Kaplan-Meier plots comparing the progression-free survival (PFS; *progression to MIBC*) with different indices of genomic alteration: tumour mutational burden (TMB), copy number burden (CNB), APOBEC enrichment, and homologous recombination deficiency (HRD). The top row is when considering all NMIBCs (*n* = 93) in the BCPP cohort, and the bottom row is considering G3pT1 patients only (*n* = 51). For each of the genomic alteration indices, patients were divided into “high” and “low” based on being >/< than the median value, respectively. In addition to the log-rank test *p*-value, multiple testing correction *p*-values are also provided on the plot

Univariate progression-free survival (PFS) analysis of all NMIBCs identified high CNB, low APOBEC mutagenesis and high HRD score as indicators of poor prognosis (adjusted *p*-value = 0.038 in each case) (Fig. 3 top row); however, these observations may arise from the higher TMB, CNB and HRD scores observed in high-grade compared to low-grade disease. Further inspecting the G3pT1 subset of tumours, low APOBEC mutagenesis and low TMB were associated with worse PFS in patients (Fig. 3 bottom row). In multivariate survival analysis comparing all the genome level aberration indices of TMB, CNB, APOBEC enrichment and HRD score, APOBEC (HR 0.31, 95% CI 0.11 − 0.88; *p*-value = 0.029) was the strongest predictor of PFS across all NMIBCs (Additional file 2: Fig. S2).

For gene-level prognostic features, univariate PFS analyses of the recurrently mutated (*n* = 37) genes in our study identified 5 candidates (*PVRL4*, *ARNT*, *RAF1*, *ERBB2* and *MDM2*) where alterations appeared indicative of worse PFS across all NMIBCs (*p* < 0.05, Additional file 2: Fig. S3); however, only *ARNT* and *ERBB2* alterations remained significant after multiple testing correction. Importantly, the prognostic effect of *ARNT* (HR 2.63, 95% 1.08–6.40; *p* = 0.034) and *ERBB2* (HR 2.90, 95% CI 1.17 − 7.20; *p* = 0.021) retained significance even on multivariate survival analysis (Additional file 2: Fig. S4).

### Transcriptome analysis

#### Expression subtyping

**Fig. 4:**
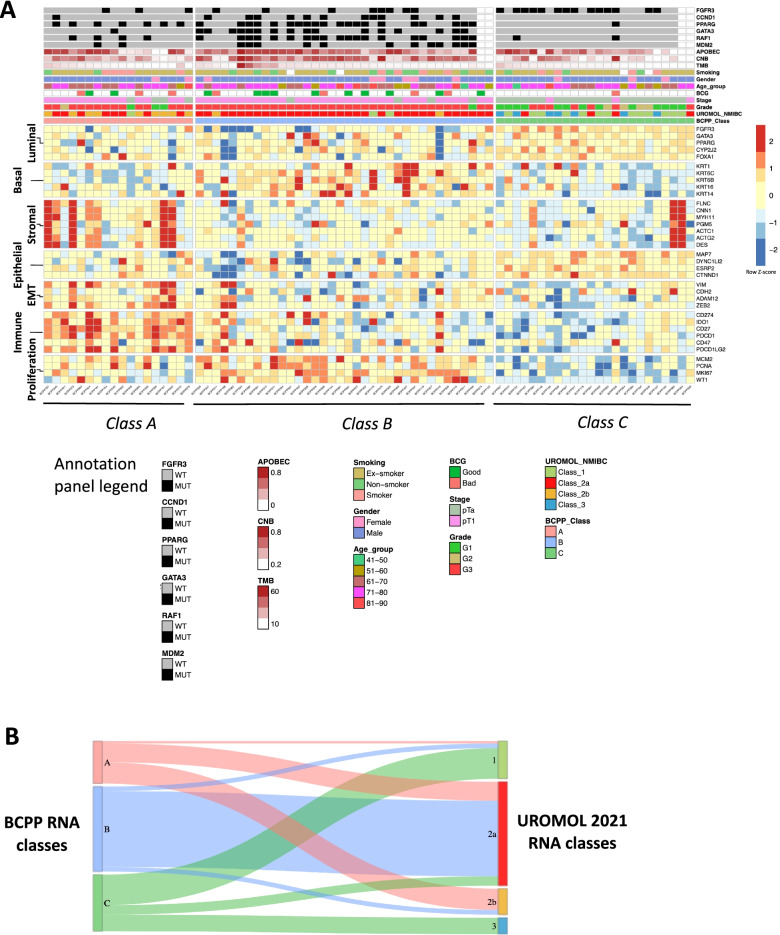
RNA sequencing samples stratified by consensus clustering. **A** Three stable expression subtypes were identified from the set of 78 RNA sequencing samples (on the *x*-axis beneath the heatmap). The characteristic traits for each of the expression subtypes were determined by the expression levels of marker genes (on the *y*-axis to the right of the heatmap). The labels for the marker gene sets are noted on the left of the heatmap. In addition, there are annotation ribbons on top of the heatmap for the clinical phenotype features (tumour grade, stage, gender, age, smoking, and BCG response) and colour coding for the expression subtypes identified in this study (“BCPP_Class”) and the subtype as assigned by the UROMOL classifier (“UROMOL_NMIBC”). **B** Sankey plot showing the sample level correspondence between the BCPP RNA classification and the UROMOL 2021 RNA classification for the same cohort of 78 RNA sequencing samples

Consensus clustering revealed three stable RNA classes (Fig. 4A and Additional file 2: Fig. S5), designated class A (*n* = 18, 78% G3, EMT gene expression), class B (*n* = 36, 97% G3, basal gene expression) and class C (*n* = 24, 71% G1/G2, luminal gene expression). Most (21 of 24; 87%) class C tumours were stage pTa, whereas pT1 stage tumours were enriched in class A (14 of 18; 78%) and class B (32 of 36; 89%).

**Fig. 5:**
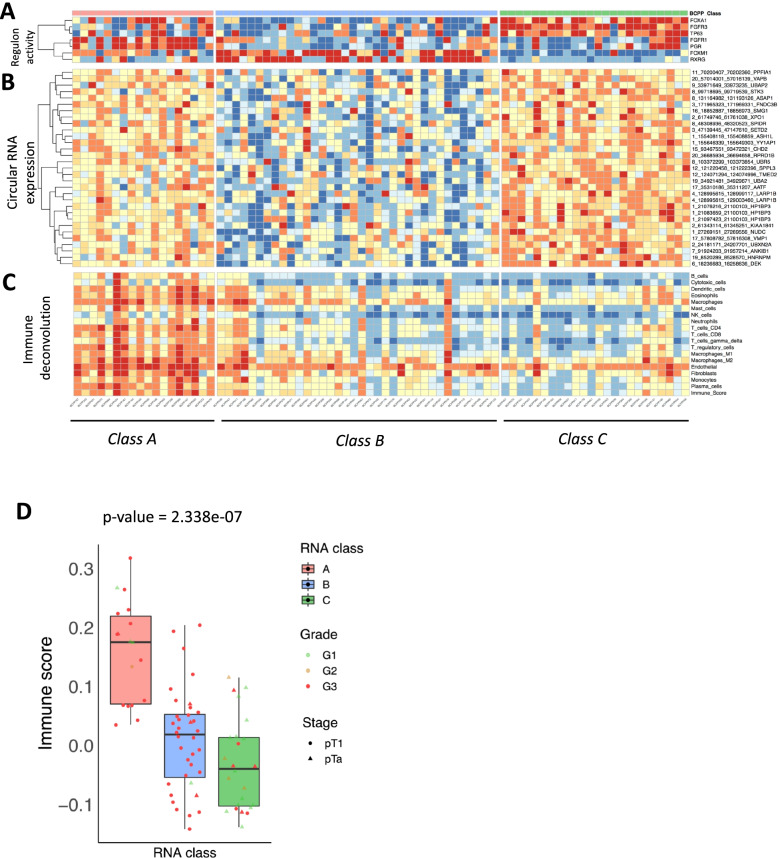
Transcriptome-wide characteristics of the expression subtypes identified. To further investigate the characteristics of the expression subtypes identified in the RNA sequencing cohort, immune deconvolution, regulon analysis, and circular RNA prediction were undertaken. **A** Regulon analysis was performed using the RTN BioConductor package, on the 23 regulons previously reported in UBC. Of the 23, seven regulons were detected with significant differential activity across expression subtypes. The regulon activity score as estimated by RTN is represented as a heatmap. **B** As the RNA sequencing was performed using total RNA, the potential impact of regulatory RNA was assessed using circular RNA (circRNA) prediction. The circular-to-linear ratio was used to perform the differential expression analysis across the expression subtypes, and 31 circRNAs were found to be statistically significant. These belong to 28 unique genes. The gene involved and the chromosomal boundaries of the back-splicing junction are given on the right-hand side. **C** Immune deconvolution was performed (using ConsensusTME) on the bulk RNA sequencing data, and the proportion of the immune cell classes estimated is denoted as a heatmap. The last row in the heatmap is for the combined “Immune_Score” as determined by ConsensusTME. **D** The contrast in the immune profile of the three expression subtypes (classes) is further highlighted in the boxplot where immune scores (as estimated by ConsensusTME) per patient are compared. Non-parametric Kruskal-Wallis test was applied to determine the statistical significance

Further, seven previously reported UBC-associated regulons [2] were active at different levels across the expression classes. *FGFR3*, *FOXA1* and *TP63* regulons had the highest activity in class C, *FOXM1* and *RXRG* had the highest activity in class B, and *FGFR1* and *PGR* had the highest activity in class A (Fig. 5A). The activity of the *FOXM1* and *FGFR3* regulons were positively and negatively correlated, respectively, with the expression of G2-M phase marker genes (*CDK1*, *UBE2C*, *TOP2A* [50]) (Additional file 2: Fig. S6; individual correlation coefficients and *p*-values provided in panel C), potentially indicating that these two regulons could have the strongest influence on cell proliferation in NMIBCs. As *FGFR3* expression (activating mutations and/or copy number amplifications) is associated with low-grade tumours (Fig. 4), *FOXM1* regulon activity could be driving the transition from low- to high-grade NMIBC. Apart from protein-coding genes, circular RNAs (circRNAs) appear to have distinctive expression across the RNA classes. ANOVA analysis identified 31 recurrent circRNAs (from 28 unique genes) discriminating between class B and classes A and C (Kruskal-Wallis adjusted *p* < 0.05), including circRNAs originating from *SETD2* and the kinase *STK3* (Fig. 5B). Thus, circRNAs could contribute to the functional molecular characterisation of UBC, as previously described [51, 52]. The immune context in tumour tissues (as estimated through immunophenotyping based on deconvolution of bulk RNA gene expression, Additional file 3: Additional Methods) indicated low levels of all major types of immune cells in class C and high immune-infiltration in class A (Fig. 5C). The composite immune score (derived from estimated levels of immune cell marker genes) was significantly different (Kruskal-Wallis *p* = 2.34 × 10e−07) when compared across RNA classes (Fig. 5D).

#### Evaluation of UROMOL 2021 NMIBC subtypes within our data

**Fig. 6:**
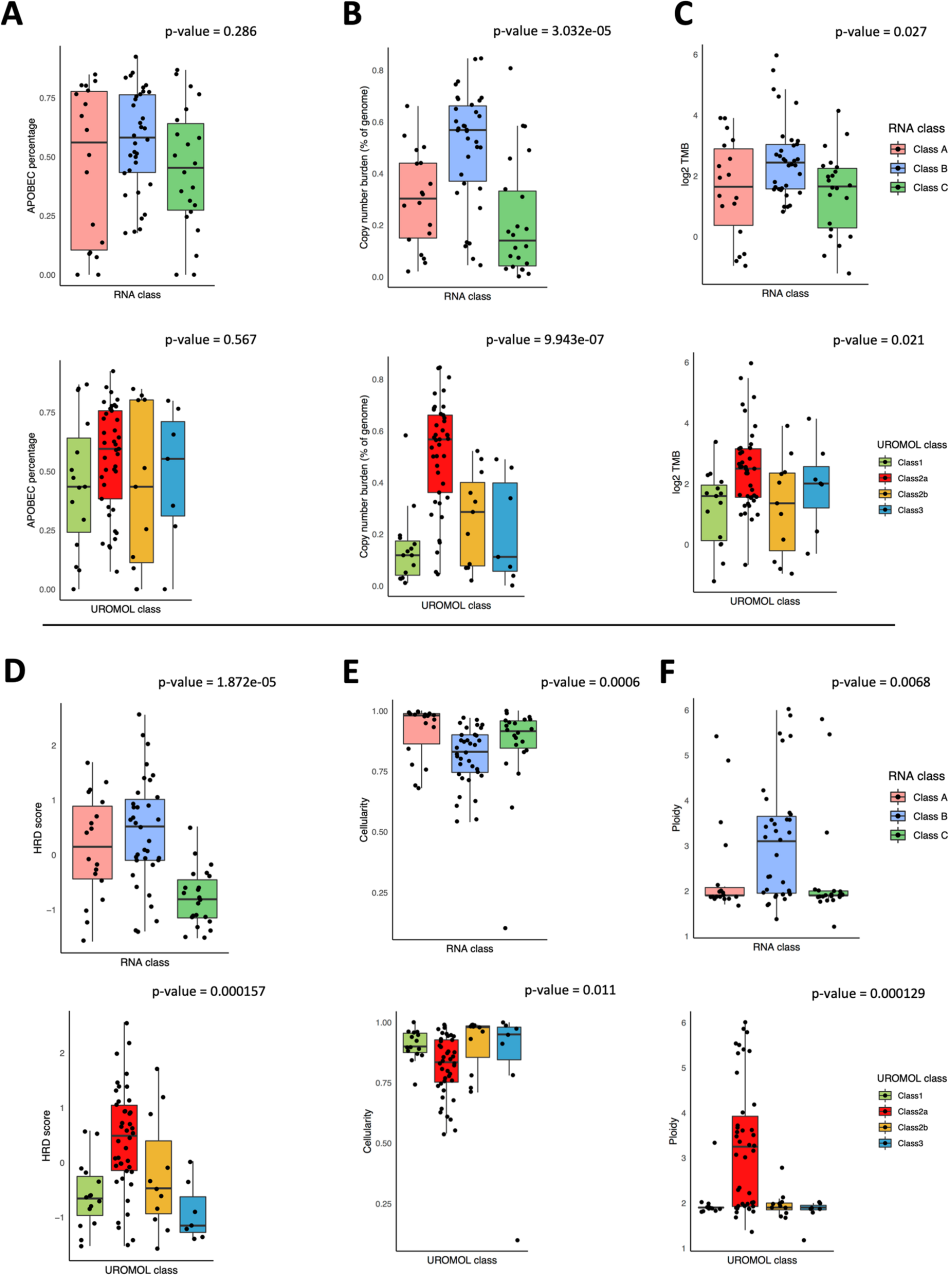
Boxplots showing the distribution of genomic alteration indices across the expression subtypes (classes). Non-parametric Kruskal-Wallis test was applied to determine the statistical significance of the differences between the RNA classes. The respective *p*-value is noted on the top-right of each boxplot. There are six panels (**A**–**F**). For each panel, the top row is the BCPP RNA class, and the bottom row is the UROMOL RNA class. The genomic alteration plotted in each panel are **A** APOBEC fraction, **B** copy number burden, **C** tumour mutational burden, **D** HRD score, **E** tumour cellularity, and **F** tumour ploidy

Our classes A, B and C show some similarities to the UROMOL 2021 NMIBC subtyping (which splits NMIBCs into classes 1, 2a, 2b and 3) [22], especially for our class B/UROMOL 2a (Fig. 4B). We used our exome data to provide additional insight into genomic events across the UROMOL subtypes: classes 1 and 3 both exhibit low CNB (Kruskal-Wallis *p*-value = 3.032e−05) and HRD score (Kruskal-Wallis *p*-value = 1.87 × 10e−05) compared to classes 2a and 2b, and between class 2a and class 2b, the former exhibits higher CNB, TMB, HRD score and ploidy (Fig. 6).

Our class A tumours are characterised by strong expression of immune marker genes (Fig. 4A); the UROMOL single-sample classifier predominantly splits them into classes 2a (8 of 18) and 2b (9 of 18) (Fig. 4B). Like our class A, UROMOL class 2b is also characterised by high expression of immune markers; the 8 samples from our class A that are classified as UROMOL 2a have somewhat lower immune marker expression.

Our class B tumours almost completely corresponded (32 of 36; 89%) with UROMOL class 2a. Our class C tumours mostly fall (20 of 24; 83%) within UROMOL classes 1 and 3; our class C and UROMOL classes 1 and 3 are characterised by *FGFR3* mutations and/or high *FGFR3* gene expression and low tumour grade/stage (Fig. 4A).

#### Molecular correlates with clinical outcomes in BCG-treated G3pT1 patients

In a pre-planned sub-study in G3pT1 patients who subsequently had received ≥ 6 induction instillations of intravesical BCG (treatment-naïve at time of sample collection), we compared those with subsequent “good” (*n* = 12) or “bad” (*n* = 8) outcomes (bad outcome defined by progression to MIBC or death from UBC within 3 years of initial TURBT; good outcome defined by the absence of progression to MIBC or death from UBC within 3 years of initial TURBT). Good outcomes were more common for class B tumours (10/14, 71%) than for class A tumours (2/5, 40%) (*p* > 0.05). Although no individual genes reached a statistically significant *p*-value (after correction for multiple testing in a differential gene expression analysis) between good and bad outcome G3pT1 NMIBCs, a gene set level analysis revealed multiple cancer hallmark gene sets that were significantly downregulated in bad outcome tumours (Additional file 1: Table S6), with interferon-alpha and interferon-gamma response pathways being most affected (adjusted *p*-value < 0.005).

### Integration of expression subtypes with genomic alterations

To identify genes differentially altered (mutations and/or copy number alterations) between expression subtypes, Fisher’s exact test was applied to the 37 genes shown in Fig. 1 (those altered ≥ 8% cases) across the three RNA classes. After multiple-testing correction, six genes were statistically significant (adjusted *p*-value < 0.05), of which one gene (*FGFR3*) was preferentially altered in class C (58% vs. 17% and 22% in class A and class B, respectively); the other five genes (*PPARG*, *RAF1*, *GATA3*, *CCND1* and *MDM2*) were preferentially altered in class B and/or class A subtypes (Fig. 4A). Of note, *PPARG* and *RAF1* are neighbours on the chr3p25.2 cytoband; hence, the majority (85%) of the copy number amplifications for these two genes co-occur. For cancer genes predominantly altered by CNVs (Fig. 1 and Additional file 2: Fig. S1), losses resulted in a tendency towards lower gene expression and gains resulted in higher gene expression (Additional file 2: Fig. S7).

Class B NMIBCs had higher CNB and TMB than classes C and A (Kruskal-Wallis *p*-value = 3.03e−05 and *p*-value = 0.027 for CNB and TMB, respectively) (Fig. 6). Class A and C NMIBCs were predominantly diploid, whereas class B NMIBCs had higher ploidy (median ploidy 3.1). The median HRD score was lowest in class C and highest in class A (Kruskal-Wallis *p*-value = 1.87e−05). Figure 6 also illustrates these alterations according to the UROMOL classification of our tumours.

### Suppressed immune pathways in poor-outcome ARNT/ERBB2-altered NMIBCs

**Fig. 7:**
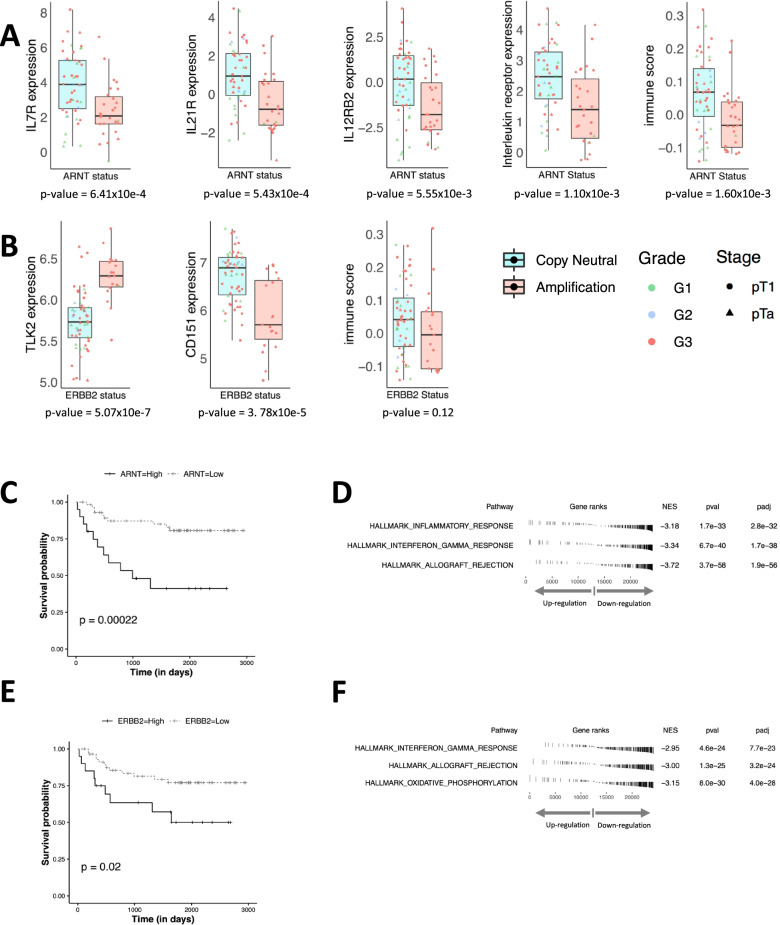
Suppressed immune phenotype in *ARNT*- and *ERBB2*-altered NMIBC patients. **A** Boxplots comparing *ARNT*-altered versus wild-type samples with Mann-Whitney *p*-values noted beneath. The first three boxplots are for the interleukin receptor genes. The fourth boxplot compares the median expression of the Gene Ontology gene set (GO: 0004907) for interleukin receptor activity. The fifth boxplot compares the ConsensusTME-derived immune score. **B** Boxplots comparing *ERBB2*-altered versus wild-type samples with Mann-Whitney *p*-values noted beneath. The first and second boxplots compare the *TLK2* and *CD151* gene expression, respectively. The third boxplot compares the ConsensusTME-derived immune score. **C**, **E** Kaplan-Meier plot comparing the progression-free survival (PFS) with *ARNT* (**C**) or *ERBB2* (**E**) expression level. The third quartile from the distribution of respective expression values (*n* = 74) was taken as the cut-off to assign “*ARNT*-High”/“*ERBB2*-High”, or “*ARNT*-Low”/“*ERBB2*-Low” samples. The depicted *p*-values are from the log-rank test. **D**, **F** The top three pathways from the gene set enrichment analysis (GSEA) in *ARNT*- (**D**) and *ERBB2*-altered (**F**) samples, respectively. “NES” is the normalised enrichment score, and the negative sign here indicates that the enrichment was found amongst the downregulated genes

We evaluated the gene expression differences of NMIBCs with and without *ARNT* and *ERBB2* amplifications (Fig. 7A, B). Amplifications of both genes resulted in their overexpression, and like the underlying gene amplification, expression of both genes was prognostic (Fig. 7C, E). Both *ARNT* and *ERBB2* amplifications are detected predominantly in high-grade NMIBCs (Fisher’s exact test *p*-value = 1.58 × 10e−2 and 2.39 × 10e−2, respectively, when comparing distribution in G3pT1 versus G1pTa). Differential gene expression (controlling for tumour grade) between *ARNT*-amplified versus samples wild-type for *ARNT* revealed downregulation of three interleukin receptors (limma based p.adj value for IL7R = 3.04 × 10e−02, IL21R = 3.95 × 10e−02 and for IL12RB2 = 2.03 × 10e−02) in *ARNT*-amplified NMIBCs (Additional file 1: Table S7). To investigate whether downregulation of interleukin receptors could be a wider phenomenon in *ARNT* amplified NMIBCs, the Gene Ontology gene set “Interleukin_receptor_activity” (GO: 0004907) was compared: median expression of interleukin receptors was found to be significantly downregulated (Mann-Whitney *p*-value = 1.10 × 10e−3) in *ARNT* amplified NMIBCs. The immune score (calculated using ConsensusTME) was also significantly lower in *ARNT* amplified samples (Mann-Whitney *p*-value = 1.60 × 10e−3).

In NMIBCs with *ERBB2* amplification, the most upregulated gene detected was *TLK2* (limma based p.adj value = 2.67 × 10e−02) (Additional file 1: Table S8) which has been reported to suppress the innate immune system [53]. *CD151* (a marker for T-cell activation [54] and/or macrophage infiltration [55]) was also downregulated (*p*-value = 3.78 × 10e−5). The ConsensusTME immune score was lower in *ERBB2*-amplified NMIBCs but was not statistically significant (Mann-Whitney *p*-value = 0.12).

Gene set enrichment analysis (GSEA) revealed immune suppression to be a transcriptome-wide phenotype through multiple MSigDb hallmark pathways. The top three enrichments in the case of *ARNT* amplification identified downregulated immune-related pathways (adjusted p-value < 10e−5) (Fig. 7D). Similarly, for *ERBB2* amplification, two of the top three enrichments identified downregulated immune-related pathways (adjusted *p*-value < 10e−5) (Fig. 7F).

## Discussion

By combining transcriptomic classification with indices of genetic aberration, we are able to add further insight into the molecular characterisation of NMIBC. Our exome and transcriptome data complement previous multi-omic MIBC studies and transcriptome-based NMIBC studies [2, 22, 56]. Overall, the findings reported herein suggest a considerable overlap between abnormal pathways in high-grade NMIBC and those in MIBC [4] (Additional file 1: Table S9). Approximately half of the somatic SNVs could be attributed to APOBEC mutational activity. Recurrent mutations were observed in genes from processes such as histone modification, cell cycle regulation and apoptosis, DNA damage repair, and RTK signalling. We find that high CNB, low APOBEC, high HRD and amplification of *ARNT* and *ERBB2* are indicators of shorter PFS across all NMIBCs. For G3pT1 NMIBCs, low APOBEC and low TMB are indicators of poor prognosis. We applied HRD scoring [57] to stratify NMIBCs, and although the use of HRD scores in UBC requires independent validation, we propose that HRD score could be used to select patients likely to benefit from PARPi or chemotherapy [58]. In addition to the most-commonly altered COSMIC Tier 1 genes, there is a long tail of both cancer-associated and other mutated genes that may play a role in urothelial carcinogenesis and require further investigation; examples include *BIRC6*, the catalytic arg-200 of *GNA13*, and Q383H in *AHR* (Additional file 1: Table S2) [59].

Consensus clustering based on gene expression identified 3 classes showing some similarity to the UROMOL 2021 subtypes [22]: our class C subtype mostly classified as UROMOL class 1 or 3, our class B subtype as class 2a and our class A subtype as class 2a or 2b. The class C subtype is typically low grade and stage NMIBCs with a diploid genome, low TMB/CNB/HRD score and APOBEC signatures, high expression of luminal marker genes and regulons, and frequent *FGFR3* mutations. The class B subtype tumours are predominantly G3 and exhibit high TMB/CNB/HRD/APOBEC signatures and expression of basal markers. The class A subtype expresses basal markers but have lower TMB/CNB/HRD score, high expression of mesenchymal genes and high levels of immune infiltration. Furthermore, the contrast between class B and C subtypes is reflected in regulon activities, with *FOXM1* and *FGFR3* regulons observed to have strong correlations with G2-M phase marker genes, but with opposite trends. This contrast may signify two ends of the spectrum of low- to high-grade NMIBC. High *FOXM1* expression has been reported to be an indicator of worse prognosis in UBC and other cancers [60–62] and our findings suggest the potential for FOXM1 inhibition therapies. Beyond protein-coding genes, potential regulatory RNAs from back-splicing events (circRNAs) demonstrate a distinctly lower level of expression in class B tumours which should be further investigated functionally (in particular, those originating from cancer-related genes) and may represent predictors of class B with future opportunities for patient stratification.

We explored the exomes and transcriptomes from G3pT1 NMIBC to identify prognostic molecular characteristics which could potentially inform clinical decisions regarding bladder preservation or cystectomy. Given that multiple combinations of environment, host genome and tumour genome may influence outcome, most studies are underpowered for patient numbers (ours included) and are influenced by treatment effects [63]. Treatment-naïve at the time of sample collection, our cohort comprises patients who have subsequently received various combinations of guideline-driven therapies and which influence outcomes; this represents a limitation. However, our exome data identified alterations in *ARNT* and *ERBB2* as poor prognostic indicators and high APOBEC and high TMB as good prognostic indicators regarding progression to MIBC. High APOBEC and high TMB are known prognostic indicators in MIBC [2] and were recently reported to be associated with good outcomes in high-grade T1 UBC by Bellmunt et al. [23]; however, *ARNT* was not included in the Bellmunt study and *ERBB2* amplifications were not reported. Importantly, we identified significant downregulation of immune-related pathways in NMIBCs with *ARNT* and *ERBB2* amplifications. This is consistent with an immune-suppressed phenotype leading to poorer outcomes in high-risk NMIBC. High *ERBB2* expression has recently been reported as a predictor of shorter RFS in pT1 disease [64], and amplification of the chr1q cytoband (where *ARNT* resides) has been reported to indicate poor prognosis in multiple cancer types [65, 66]. Although *ARNT* amplification could be a proxy for chr1q amplification (and other genes in this region, such as *SETDB1*, might functionally contribute to prognostic differences), it is an interesting candidate for future investigation: it is the only COSMIC cancer gene within the amplified region, germline polymorphisms in *ARNT* are associated with an increased risk of bladder cancer [67], and the protein encoded by *ARNT* dimerises with the protein encoded by the *AHR* gene which is recurrently mutated in UBC [59]. Other genetic alterations identified as significant prognostic indicators by Bellmunt [23] were not significant in our cohort. The results of our analyses should be interpreted in the context of the limited size of our cohort, and the observed variability between our and previous studies suggests that larger patient numbers are required to determine robust prognostic indicators. Hence, re-analysis of combined data from all sequencing studies of G3pT1 tumours with available treatment and outcome data could be worthwhile. We had limited capability for detection of sub-clonal events in UBC, due to a lack of multi-region sampling and/or longitudinal sampling in the study cohort. Nevertheless, the estimated median tumour cellularity was > 85%, and so we are confident of the recurrent somatic aberrations identified and the transcriptome-based sub-typing undertaken. We also investigated potential markers for BCG treatment response, albeit for a small subset of high-grade (G3pT1) samples; we identified differentially regulated immune-related pathways (interferon-alpha and -gamma response pathways) that warrant further investigation. This finding, along with TMB being a poor prognostic indicator and the downregulation of immune-related pathways in poor-prognosis *ARNT-* and *ERBB2*-amplified NMIBCs, suggests that the immunological properties of NMIBCs are likely important determinants of future progression.

## Conclusions

We have presented the largest combined exome and transcriptome analyses of NMIBC to date. Our data confirm that low APOBEC signature is a poor prognostic indicator in the tumours from NMIBC patients, including those with G3pT1 disease and that high expression of interferon response pathways may be indicative of either good prognosis or good response to BCG therapy. An immune-suppressed phenotype associated with *ARNT* and *ERBB2* amplification is associated with poorer progression-free survival and we highlight these genes as candidates for further studies. We have also identified differences in *FGFR3* activation and immune marker expression between our transcriptome classes and those of UROMOL 2021, highlighting the importance of multi-omic profiling in accurately classifying NMIBCs.

## Supplementary Information

**Additional file 1: Readme.** Index page for the constituent tables in this file. **Table S1.** Clinical phenotype for the 96 patients in the BCPP cohort. **Table S2.** Somatic mutations: Single Nucleotide variants (SNVs) and Insertion/ Deletions (InDels) identified from Whole-exome sequencing (WES). **Table S3.** All mutated genes ordered by their mutation recurrence count. **Table S4.** GISITIC identified focal Copy Number Amplification peaks. **Table S5.** GISITIC identified focal Copy Number Deletion peaks. **Table S6.** MSigDB (hallmark gene sets) for genes differentially expressed in Bad versus Good outcome in G3pT1 tumours. **Table S7.** Differentially expressed genes in ARNT altered versus samples wild-type for ARNT. **Table S8.** Differentially expressed in ERBB2 altered versus samples wild-type for ERBB2. **Table S9.** Genomic alteration recurrence frequencies as identified in NMIBC (this cohort) and TCGA MIBC (as seen in cBioPortal).

**Additional file 2: Fig. S1.** Focal copy-number alteration peaks identified in NMIBC. **Fig. S2.** Multivariate comparison of genomic alteration indices with progression-free survival (PFS). **Fig. S3.** Kaplan-Meier plots comparing progression free survival (PFS) for patients altered (mutated and or CNA) versus wild-type for specific genes. **Fig. S4**. Multivariate comparison of altered (mutated and/ or CAN) genes with progression-free survival (PFS). **Fig. S5.** Consensus clustering workflow. **Fig. S6.***FOXM1* regulon activity positively correlates with cell proliferation. **Fig. S7.** Effect of copy number alteration on gene expression.

**Additional file 3: Additional Methods.** Extended details on Materials and Methods.

## Data Availability

Whole-exome sequencing and RNA sequencing data for the bladder cancer patients from this study have been submitted to the European Genome-Phenome Archive (EGA) (https://ega-archive.org/) and will be publicly available under controlled access under accession codes: EGAS00001006110 (https://ega-archive.org/datasets/EGAS00001006110) [32] and EGAS00001004358 (https://ega-archive.org/studies/EGAS00001004358) [31], respectively.
