# Supplementary material for: Combined exome and transcriptome sequencing of non-muscle-invasive bladder cancer: associations between genomic changes, expression subtypes, and clinical outcomes
Source: Genome Med. 2022 Jun 3;14:59. doi: 10.1186/s13073-022-01056-4 (PMC9164468; doi:10.1186/s13073-022-01056-4)
Supplement: Supplementary file 2 — Additional file 2: Fig. S1. Focal copy-number alteration peaks identified in NMIBC. Fig. S2. Multivariate comparison of genomic alteration indices with progression-free survival (PFS). Fig. S3. Kaplan-Meier plots comparing progression free survival (PFS) for patients altered (mutated and or CNA) versus wild-type for specific genes. Fig. S4. Multivariate comparison of altered (mutated and/ or CAN) genes with progression-free survival (PFS). Fig. S5. Consensus clustering workflow. Fig. S6.FOXM1 regulon activity positively correlates with cell proliferation. Fig. S7. Effect of copy number alteration on gene expression. [file 13073_2022_1056_MOESM2_ESM.pdf]

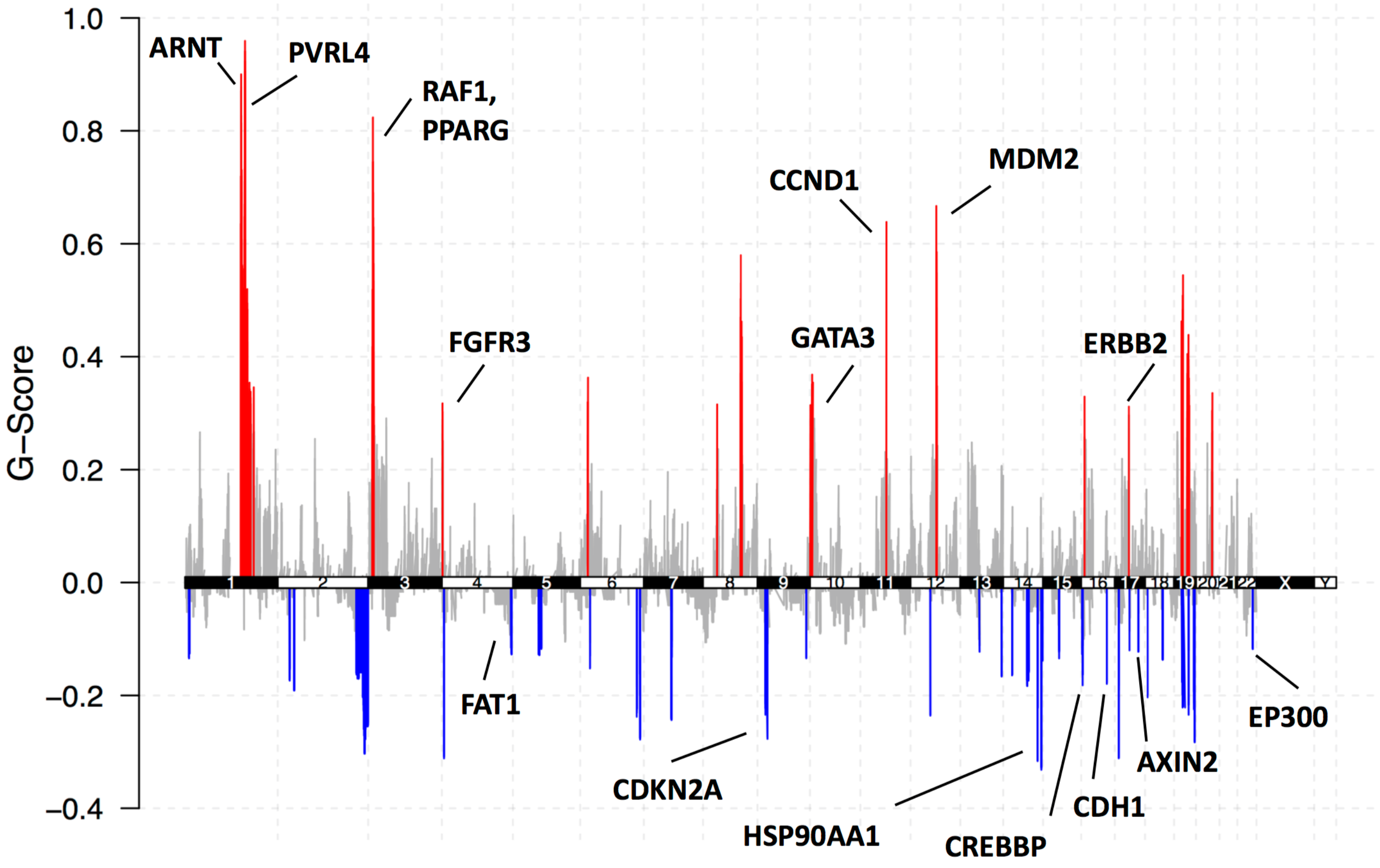

<u>Figure S1</u>: Focal copy-number alteration peaks identified in NMIBC.
The x-axis shows the chromosomes in alternating bands of black and white. The y-axis is the GISTIC score (defined as the negative logarithm of the probability of observing a candidate copy-number event with given amplitude and frequency, given the background copy-number alteration rate). The score is positive for copy-number amplification (red) and negative for copy-number deletion (blue) peaks. The peaks in grey did not achieve statistical significance. Genes mapping to significant peaks, and which are also present in COSMIC Cancer Gene Census, have been labelled.

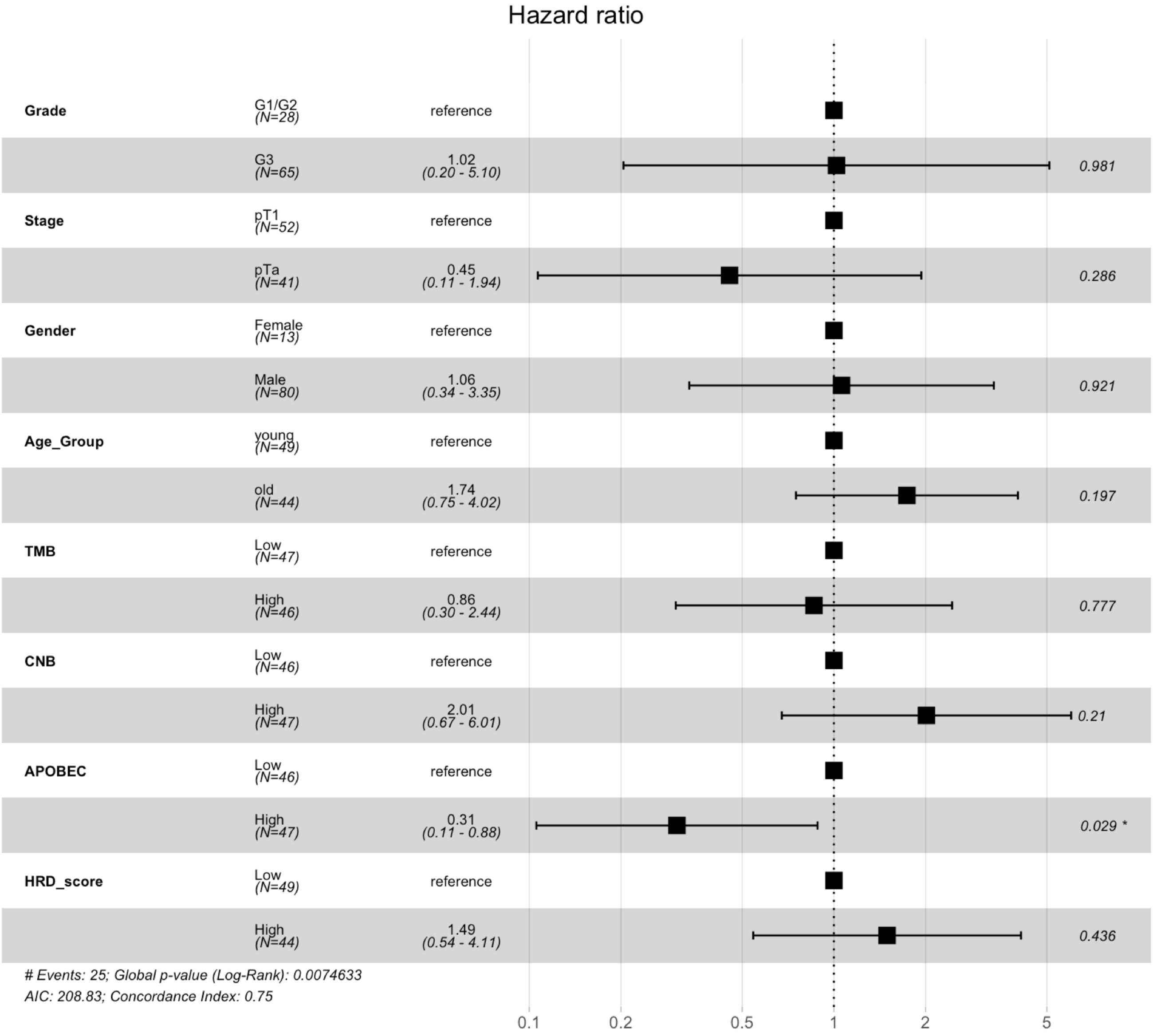

Figure S2: Multivariate comparison of genomic alteration indices with progression-free survival (PFS).

In addition to the genomic alteration indices, tumour grade and stage are also considered to test if significance is retained even after those two clinical phenotypes are considered. The dotted perpendicular line from hazard ratio (HR) 1 is indicative of no effect on PFS. Significant deviation from HR value of 1 is indicated by p-value < 0.05. The patients were grouped into 'High' or 'Low' depending on being >/< the median value. 'TMB' is for tumour mutational burden, 'CNB' is for copy number alteration burden', 'APOBEC' is for APOBEC enrichment and 'HRD_score' is homologous recombination deficiency score.

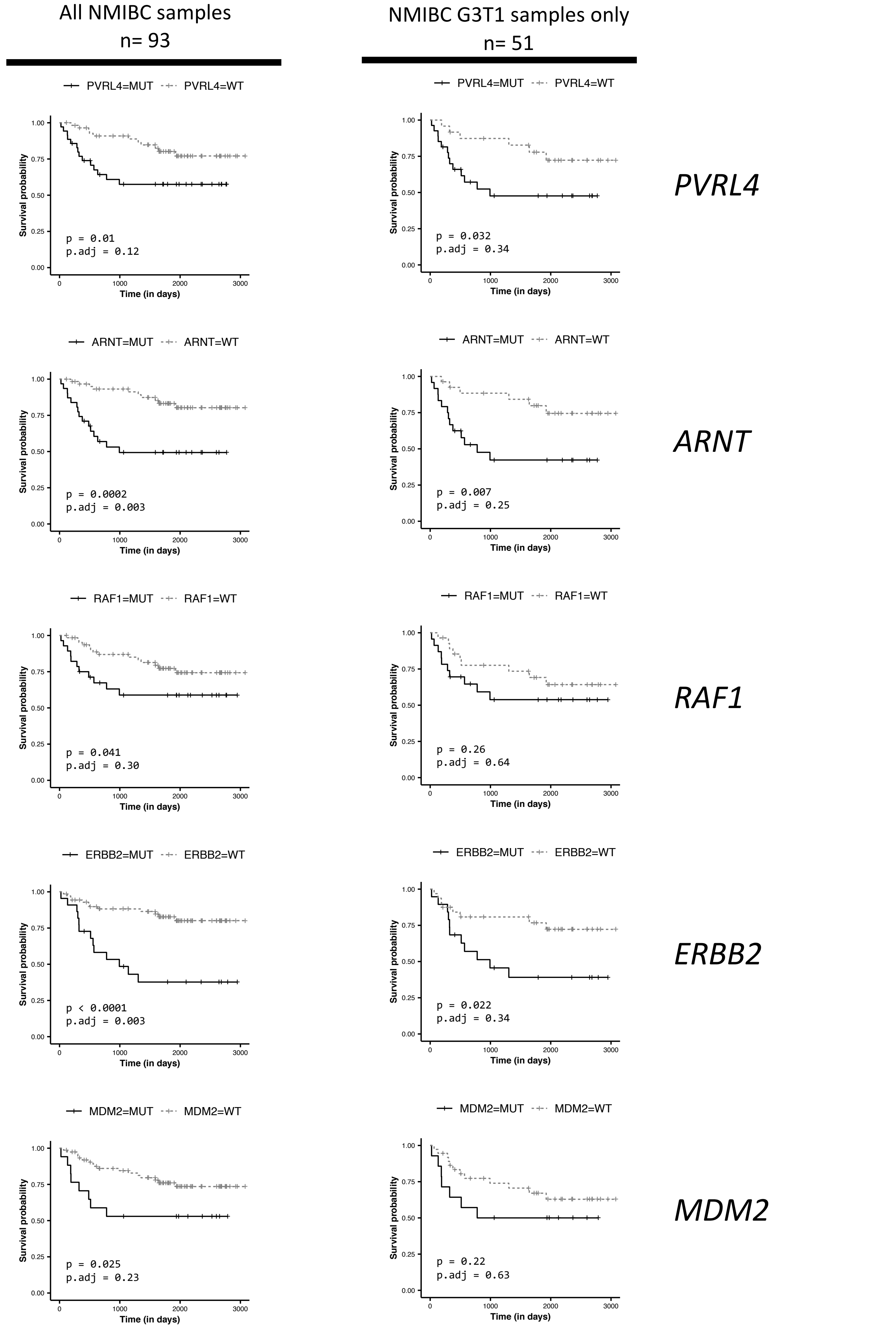

Figure S3: Kaplan-Meier plots comparing progression free survival (PFS) for patients altered (mutated and or CNA) versus wild-type for specific genes.
The plots on the left consider the total set of NMIBC patients (n=93), whereas those on the right consider G3pT1 patients (n=51) only. Each pair of plots is for a specific gene.

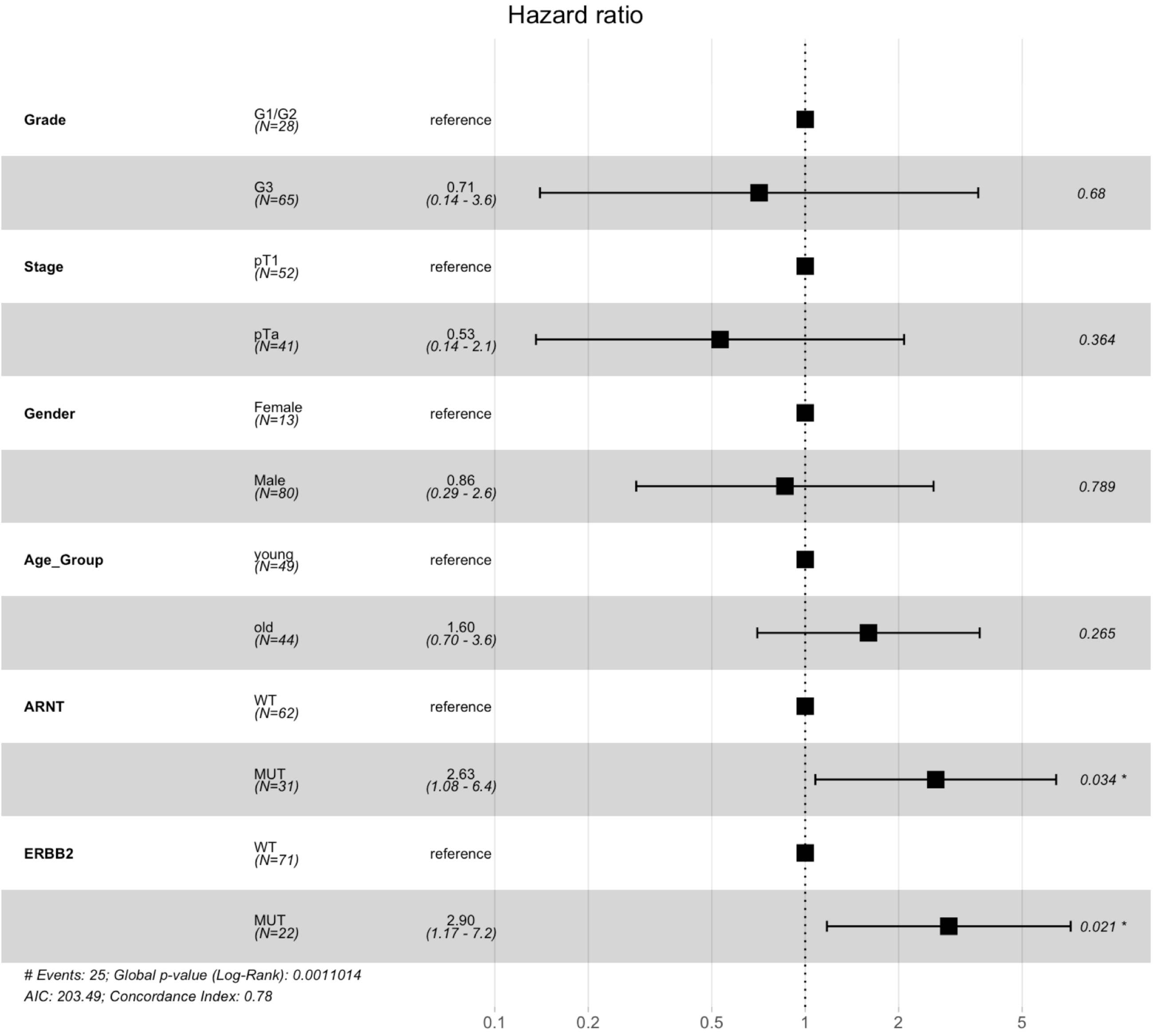

<u>Figure S4</u>: Multivariate comparison of altered (mutated and/ or CNA) genes with progression-free survival (PFS).
In addition to the genes, tumour grade, stage, gender and age are also considered to test if significance is retained after these four clinical phenotypes are considered. The dotted perpendicular line from hazard ratio (HR) 1 is indicative of no effect on PFS. Significant deviations from HR value of 1 are indicated by p-value< 0.05.

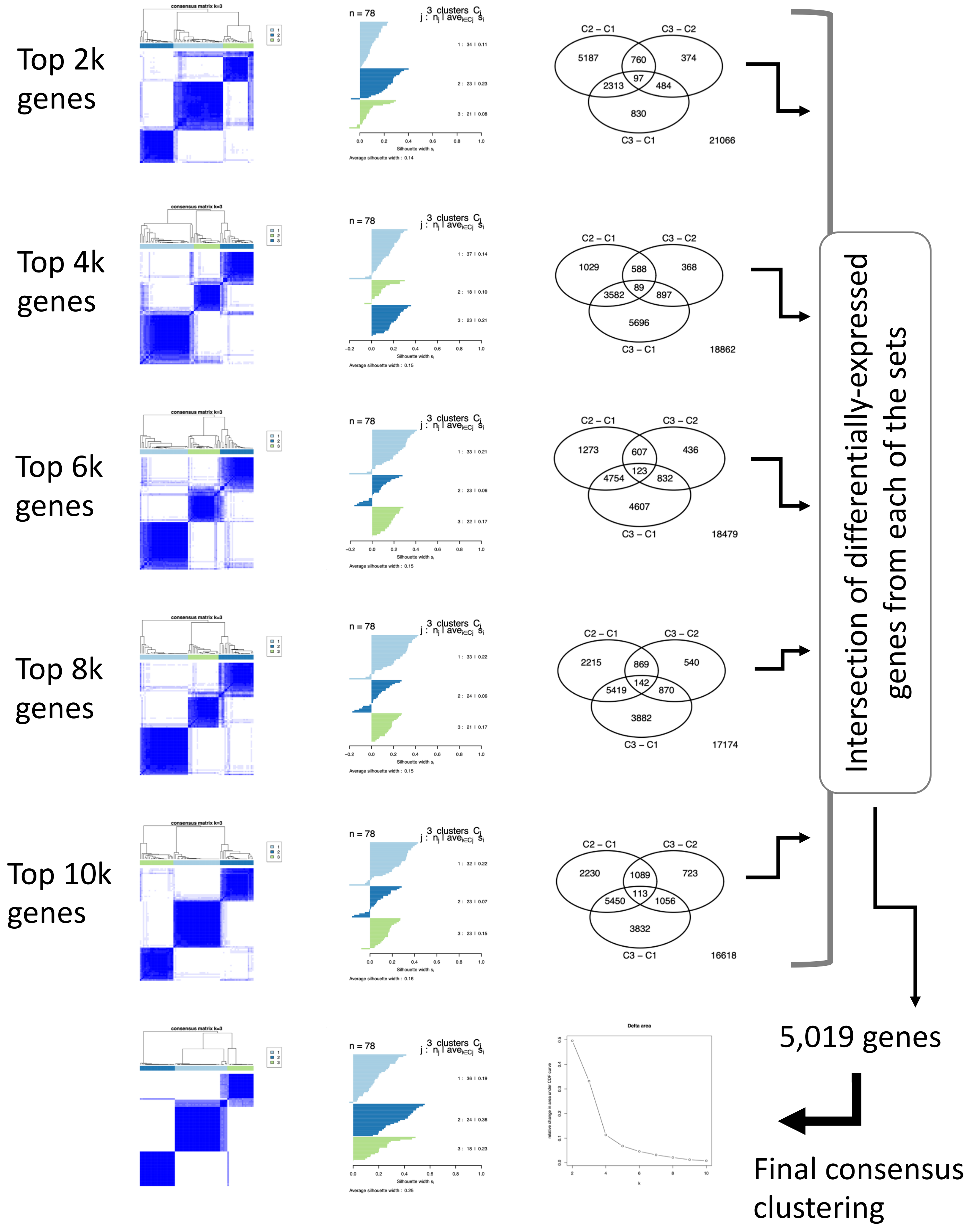

Figure S5: Consensus clustering workflow.

Consensus clustering was applied to increasing subsets (starting from 2,000 to 10,000, in steps of 2,000) of top variable (in terms of median absolute deviation) genes. In each case, k=3 was the optimal solution as per the delta area plot. For the k=3 solution, silhouette widths were calculated for each of the samples. Those samples with positive silhouette width score were then taken to perform limma-based differential gene expression as per the cluster membership. Once this process was completed for all the five sets (2k, 4k, 6k, 8k and 10k), an intersection was taken to find the genes which appeared differentially expressed in all the sets. This gene set (8,807 genes) was then further filtered for genes which had raw count ≥5 in ≥8 samples, resulting in 5,019 genes. This gene set was then the input for the final consensus clustering. The cluster membership thus derived has been used for downstream comparisons with clinical and molecular phenotype traits.

A

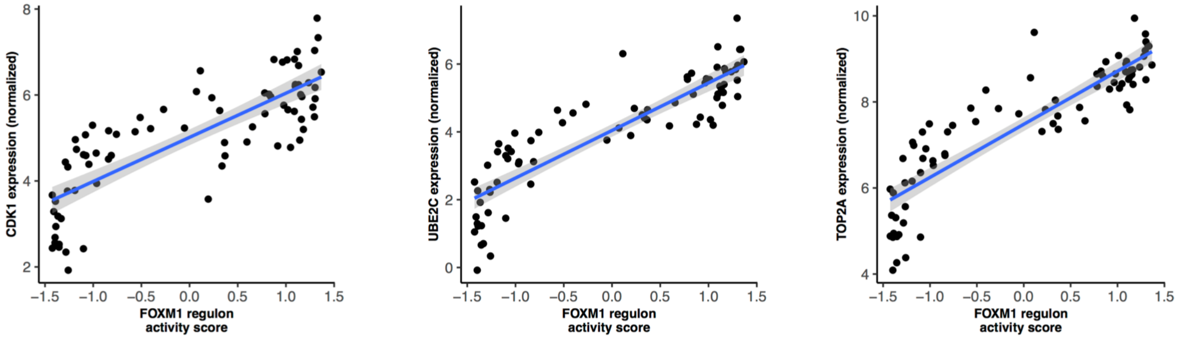

B

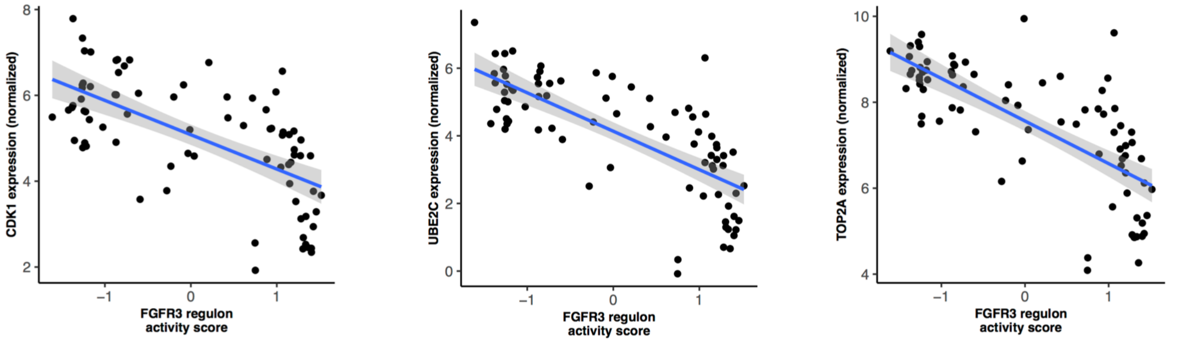

C

		Regulons						
		**FOXM1**	**FGFR3**	**FGFR1**	**FOXA1**	**PGR**	**RXRG**	**TP63**
Cell-cycle genes	**CDK1**	0.80***	-0.64***	0.02	-0.25*	-0.22	0.21	-0.35*
	**UBE2C**	0.88***	-0.73***	-0.02	-0.36*	-0.30*	0.27*	-0.49***
	**TOP2A**	0.88***	-0.73***	-0.03	-0.37**	-0.30*	0.29*	-0.46***

Figure S6: ***FOXM1* regulon activity positively correlates with cell proliferation.**
Genes *CDK1*, *UBE2C* and *TOP2A* are known to have peak expression during the G2-M phase (cell division) of the cell cycle. FOXM1 regulon activity shows consistently high positive correlation with expression level of all the three genes (Panel **A**). Conversely, activity of the *FGFR3* regulon is consistently negatively correlated with expression levels of the cell-cycle (G2-M phase) genes (Panel **B**). Panel **C** shows the correlation coefficient score (Pearson) and the p-value (*** for $<10^{-5}$, ** for $<10^{-3}$ and * for <0.05) for all the nine regulons detected in the NMIBC RNA-seq cohort. Correlation scores >0.6 or, <-0.6 are highlighted in red or blue respectively.

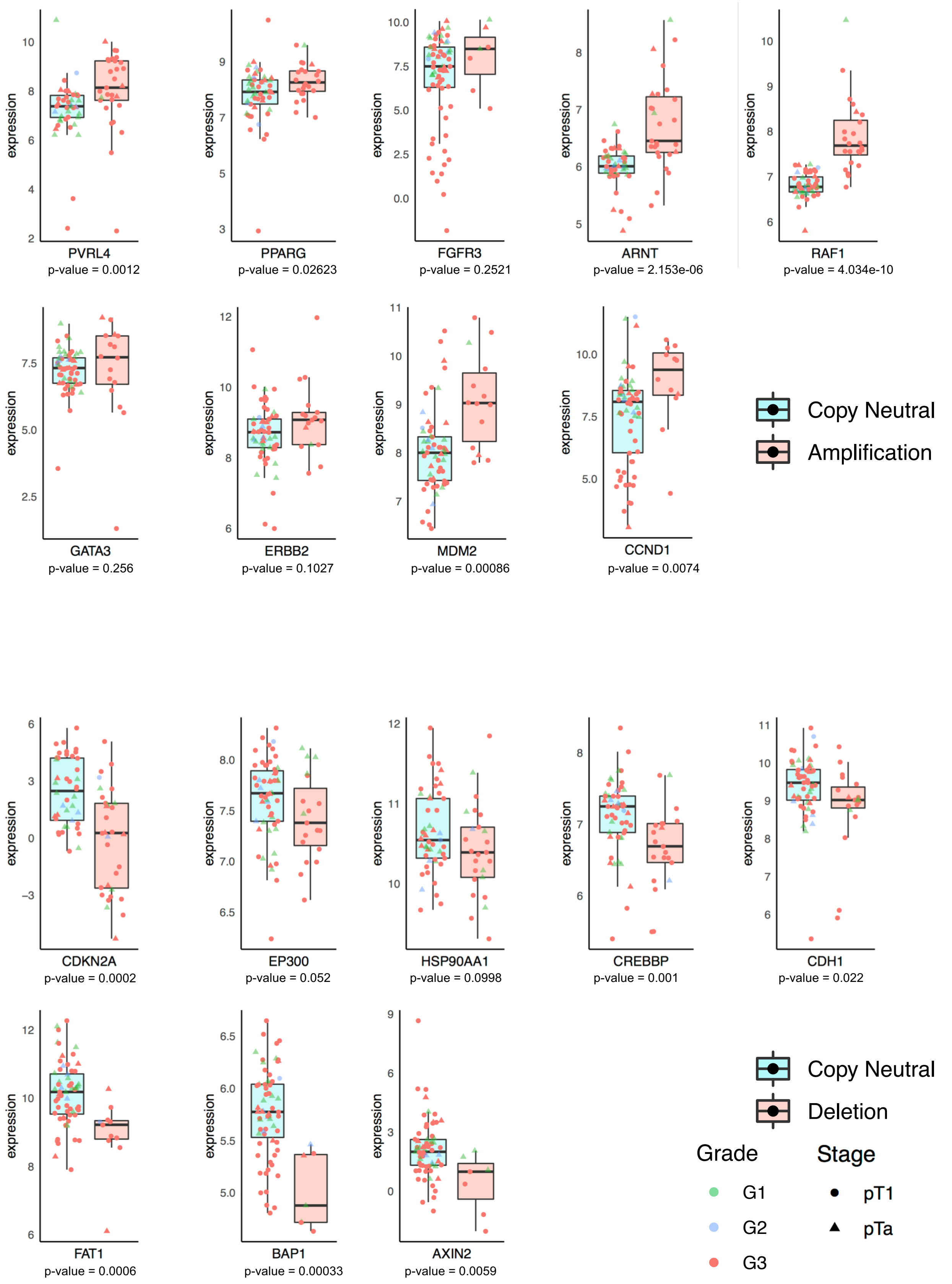

<u>Figure S7</u>: Effect of copy number alteration on gene expression.
Of the 37 genes in OncoPlot, those with statistically significant CNA (Copy Number alteration) (n=17) were tested for downstream effects on gene expression by performing Mann-Whitney two-tailed tests between samples with CNA versus those copy neutral for that respective gene. The p-values are noted beneath the x-axis in each case. The y-axis denotes the normalised expression value for that respective gene.
