# Supplementary material for: Combined exome and transcriptome sequencing of non-muscle-invasive bladder cancer: associations between genomic changes, expression subtypes, and clinical outcomes
Source: Genome Med. 2022 Jun 3;14:59. doi: 10.1186/s13073-022-01056-4 (PMC9164468; doi:10.1186/s13073-022-01056-4)
Supplement: Supplementary file 3 — Additional file 3: Additional Methods. Extended details on Materials and Methods. [file 13073_2022_1056_MOESM3_ESM.docx]

**Combined exome and transcriptome sequencing of non-muscle-invasive bladder cancer: associations between genomic changes, expression subtypes and clinical outcomes**

Anshita Goel**†**^1,2^, Douglas G Ward**†**^1,2^, Boris Noyvert**†**^2,3,4^, Minghao Yu^1,2^, Naheema S Gordon^1,2^, Ben Abbotts^1,2^, John K Colbourne^5^, Stephen Kissane^5^, Nicholas D James^6,7^, Maurice P Zeegers^8,9^, KK Cheng^10^, Jean-Baptiste Cazier^2,3^, Celina M Whalley^11^, Andrew D Beggs^2,11^, Claire Palles^2^, Roland Arnold**‡**^1,2^, Richard T Bryan**‡**^1,2^.

**†** Authors contributed equally.

**‡** Authors contributed equally.

**Affiliations**

1: Bladder Cancer Research Centre, University of Birmingham, Birmingham, UK.

2: Institute of Cancer and Genomic Sciences, University of Birmingham, Birmingham, UK.

3: Centre for Computational Biology, University of Birmingham, Birmingham, UK.

4: CRUK Birmingham Centre, University of Birmingham, Birmingham, UK.

5: School of Biosciences, University of Birmingham, Birmingham, UK.

6: Institute of Cancer Research, London, UK.

7: The Royal Marsden NHS Foundation Trust, London, UK.

8: Department of Complex Genetics and Epidemiology, School of Nutrition and Translational Research in Metabolism, Maastricht University, Maastricht, The Netherlands.

9: CAPHRI School for Public Health and Primary Care, University of Maastricht, Maastricht, The Netherlands.

10: Institute of Applied Health Research, University of Birmingham, Birmingham, UK.

11: Genomics Birmingham, University of Birmingham, Birmingham, UK.

**Corresponding authors**

Richard T Bryan, Bladder Cancer Research Centre, University of Birmingham, Birmingham, UK. [r.t.bryan@bham.ac.uk](mailto:r.t.bryan@bham.ac.uk), +44 121 414 7870.

Roland Arnold, Bladder Cancer Research Centre, University of Birmingham, Birmingham, UK.

r.arnold.2@bham.ac.uk

**Additional Methods**

**Patient sample collection**

Fresh frozen tumour specimens and paired blood samples were collected as part of the Bladder Cancer Prognosis Programme (BCPP, ethics approval 06/MRE04/65). Patients were recruited consecutively from 2005 to 2010 from ten hospitals in the West Midlands (UK), and gave informed consent for enrolment based upon initial cystoscopic findings suggestive of primary UBC (Urothelial Bladder cancer). All patients were newly diagnosed and treatment-naïve at biospecimen collection and were subsequently treated and monitored according to contemporary European Association of Urology (EAU) guidelines (including re-resection where indicated) and EAU risk groups (for NMIBC). Inclusion and exclusion criteria are detailed elsewhere. Where necessary, tumour grade and stage records were amended according to results of early re-resection or cystectomy. We used the 1973 grade classification as it was in universal use in the UK at the time of patient recruitment, is the basis for the EORTC and EAU NMIBC risk tables and has comparable utility to the 2004/2016 classification. All included tumours were purely or predominantly transitional cell carcinomas. Patient demographics are shown in **Additional file 1:** **Table S1**. Tissues were collected at transurethral resection (TURBT), snap-frozen, and stored at -80°C. During follow-up 32 of the 96 patients died. In NMIBC patients, recurrence occurred in 50 cases, progression to MIBC in 26 cases, and UBC was recorded as the cause of death in 17 cases. Tissues and blood were stored at -80°C; DNA was extracted from 25mg tissue and 100μl paired blood using DNeasy Blood and Tissue kits, and RNA from 25 mg frozen tissue using RNeasy kits (Qiagen, Hilden, Germany).

**Library preparation and sequencing**

Of 96 tumours, 93 underwent exome sequencing (with paired germline DNA) and 78 underwent RNA sequencing, with a total of 75 overlapping sequencing datasets. Sequencing libraries were prepared using Nextera® Rapid Capture Exome and TruSeq® Stranded RNA LT kits (Illumina, San Diego, USA) and HiSeq/NextSeq sequenced. The TERT promoter and the 5’ end of exon 7 of FGFR3 were sequenced separately using amplicon-based library preparation as they were not targeted/not captured well during exome library preparation.

**Sample pair identity**

To confirm that the tumour and paired germline exome sequencing and the corresponding RNA-sequencing sample belonged to the same individual, we compared variant allele read frequencies (proportion of reads carrying the non-reference allele) at hundreds of common SNPs (population-level minor allele frequency greater than 0.2) across the genome that had good coverage in all the samples. We used mean difference in variant read frequencies between 2 samples as a distance measure.

**Sequence data processing**

**Whole Exome Sequencing (WES)**

**Quality control (QC)**

Raw fastq files were checked with FastQC (version 0.11) for per base Phred score quality distribution and presence of adapter sequences. Subsequently, adapter sequences if present, were clipped and low-quality stretches from read ends were trimmed using Cutadapt (version 1.1) **(1)** and Trimmomatic (version 0.36) **(2)**, respectively. Only those read pairs were retained where both mates had >=35 bases remaining after adapter clipping and quality-based trimming.

**Alignment and mapping**

QC processed reads were mapped to the human genome (version GRCh37) using the *mem* algorithm of the Burrows-Wheeler Aligner (BWA version 0.7.15) **(3)** to obtain SAM (Sequence Alignment and Mapping) format files, which were then coordinate sorted, converted to binary (BAM) format and flagged for PCR duplicates using Picard tools (version 1.14.0)**.** The resultant BAM files were then put through local realignment (using InDels and SNPs from 1000Genomes) and base quality score recalibration, using GATK (Genome Analysis Tool Kit; version 3.6). The final BAM files thus obtained were used for calculating depth of sequencing coverage statistics and were the starting point for mutation calling.

**Somatic mutation calling, filtering and variant annotation**

Somatic SNVs (Single Nucleotide Variants) and Indels (Insertions/ Deletions) were identified using MuTect2 and GATK4 (version 4.1.4.0) **(4)** on each of the tumour-normal pairs separately as following. A panel of normals (PoN) was created using the germline samples (GATK tool CreateSomaticPanelOfNormals). Then MuTect2 (version 2.2) was run for each of the tumour-normal pairs using the PoN as well as gnomAD (Genome Aggregation Database v2) **(5)** VCF as a germline resource. The call set thus obtained was further filtered (GATK tool FilterMutectCalls) using contamination estimates and read-orientation model metrics. After selecting for ‘PASS’ calls only in the somatic VCF (Variant Call Format) thus obtained per tumour-normal pair, additional filtering was applied on quality metrics of TLOD (Log10 likelihood ratio score of variant existing versus not existing), CONTQ (Phred-scaled qualities that alt alleles are not due to contamination), ROQ (Phred-scaled qualities that alt alleles are not sequencing errors), GERMQ (Phred-scale quality that alt alleles are not germline variants) and STRANDQ (Phred-scaled quality of strand bias artifact) using empirically determined thresholds. Finally, each of the calls had to clear read-depth support thresholds of >=10 overall for the variant locus in both the tumour and the paired germline sample and additionally >=5 for the alternate allele specifically in the tumour sample.

Variant Effect Predictor (VEP version 94 cache on GRCh37) **(6)** was used to annotate the somatic SNVs and Indel calls, using additional annotation datasets of gnomAD genomes-based population frequencies and dbscSNV (database of splicing-consensus SNVs) predictions. Variants were designated as polymorphic loci and filtered off if present in any of the population datasets of 1000genomes, HapMap, ExAC or gnomAD at frequency of 10^-3^ or higher. Candidate genes affected were identified if the variant impact was classified as HIGH or MODERATE, or if predicted to be splice-altering in dbscSNV.

**Distribution of somatic mutation genes across cellular pathways**

The gene sets provided in the TCGA-MIBC 2017 publication were used to determine the alteration frequency of RTK/RAS/PI3K, histone modification, TP53/ Cell cycle, DNA damage repair and cohesion complex pathways in our NMIBC cohort.

**Mutational signature analysis**

The total set of somatic SNVs identified per tumour-normal pair was used for estimation of the six-base substitution (C > A/G/T and/or T > A/C/G) frequencies using the R package deconstructSigs (version 1.8.0) **(7)** to predict contribution from each of the 30 mutational signatures (version 2) from COSMIC, per tumour sample. Using the estimated percentages extracted for the signature types per sample, hierarchical clustering was performed using ward.D2 method implemented in R.

**Copy number analysis**

CNVkit (version 0.8.3) **(8)** was used on paired tumour-normal samples. BAM files from whole exome sequencing to estimate tumour-specific genome wide copy number changes. Briefly, somatic log2 copy number ratio was estimated and segmentation algorithm applied to infer discrete copy number segments for each tumour sample. Two outlying tumours with extremely high numbers of segments were excluded. GISTIC (version 2.0.23) **(9)** was then used to identify focal copy number peaks in the tumour exome. After examining the output for potential false positives when ran using default parameters, GISITC was given thresholds of 0.3 for both amplification and deletion to identify the final peaks. The results from GISTIC on tumour samples, were imported into maftools (v1.4.28) **(10)** in the R environment, for tabulating and plotting the results at the gene level and focal copy number peak level. The effect of CNVs on gene expression was evaluated by comparing gene expression levels in tumours with or without copy number gain or loss.

**Tumour mutational burden (TMB) and Copy-number burden (CNB)**

For each sample, the total number of somatic nucleotide variants (SNVs) were divided by the total number of bases sequenced (at >=10x read depth with mapping quality score >= 30 and phred score quality >=20), to get the value of mutation count per sequenced base. This value was expressed in per Megabase to derive the tumour mutational burden. For copy-number burden calculation, the total size of segments with mean value >=0.1 or, <= -0.1 were calculated per sample and divided by the total size of the autosomal genome (chrX and chrY were excluded) to derive the fraction of genome under copy-number.

***In-silico* estimation of tumour purity**

Tumour cellularity estimation as a surrogate marker for tumour purity, was done using Sequenza (version 3.0.0) **(11)** method by comparing somatic variant allele frequencies and the log-ratio of read-depth in tumour versus the paired normal WES. Briefly, homozygous and heterozygous position and read-depth ratio information are extracted from the alignment data in genomic window bins of 50bp along with GC content normalisation. A probabilistic model is then fit to estimate the tumour cellularity (or, purity) and overall ploidy on a per sample basis.

**Homologous recombination deficiency (HRD) estimation**

The three indices of HR deficiency: Telomeric Allelic Imbalance (HRD-TAI), Loss-Of-Heterozygosity profiles (HRD-LOH) and Large-scale State Transitions (HRD-LST) were estimated per tumour sample using scarHRD package **(12)** on the processed output from Sequenza. To create a homogenized score from the sum of the scores of the three indices, z-score transformation was applied.

**RNA Sequencing**

**Alignment and expression quantification**

QC processed reads were mapped to the human reference genome (GRCh37) and the transcriptome (GTF annotation reference Ensembl rel.87) using STAR aligner (ver. 2.5.2b) **(13)**. Gene level count data was obtained for each of the 78 samples using the –quantMode option in STAR.

**Normalization and differential expression**

Gene level raw read counts from all samples were combined and used as input to limma package in R (ver. 3.4.0), where the data was normalised using the voom method **(14)** which performs variance stabilisation and returns log-transformed normalized count values. These values were then used for differential expression comparisons using the empirical Bayes method within the limma (version 3.44.1) package.

**Functional enrichment analysis**

To further assess function of differentially expressed genes (DEGs), we carried out gene set enrichment analysis using the Enrichr online tool **(15)**. The biological processes affected by the DEGs (adjusted p-value cutoff 0.05) were adjudged based on enrichment in gene sets of KEGG (Kyoto Encyclopedia of Genes and Genomes) pathways. We also used a cutoff-free approach implemented in GAGE **(16)** R Bioconductor package (version 2.36.0) to compare the differential expression of genes in a given set to the background of all genes. We applied the tool to the MSigDB hallmark gene set collection available from https://www.gsea-msigdb.org/gsea/msigdb/collections.jsp.

**Genetic alteration versus gene expression**

Samples altered for either ARNT or ERBB2, were compared with the samples wild-type for the respective gene. Differential gene expression using R package limma (v.3.50.0), was assessed with adjustment for tumour grade and at adjusted (Benjamini-Hochberg) p-value threshold of < 0.05. Gene set enrichment analysis was performed using fgsea (**17**) R BioConductor package (v.1.20.0), on genes ranked by the t-statistic from the differential expression output. MSigDb (v.7.5.1) hallmark pathway set (n=50 pathways) was examined using fgsea and pathways with normalised enrichment score (NES) >=2 and adjusted p-value < 0.01 were considered significant.

**Consensus clustering**

Stratification of the BCPP RNA-seq samples based on consensus clustering was performed using the ConsensusClusterPlus (version 1.50) **(18)** implemented in R. Briefly, increasing sets of the most variable genes (as per median absolute deviation across samples) were taken to perform consensus clustering to identify the optimal k (number of clusters) each time. Further, for the optimal k, only samples with positive silhouette width were considered to perform differential gene expression using limma (version 3.42) package, among the clusters. Doing so for gene sets from 2,000 to 10,000 (in steps of 2,000), the intersection set was identified that was differentially expressed every time and the constituent genes further filtered to be above baseline expression level (raw count >=5 in >=8 samples). This set of 5,019 genes across the 78 samples was again subjected to consensus clustering for the final assignment of cluster membership.

**Regulon analysis**

The activity level of the 23 regulons previously reported in UBC **(19)**, was assessed in the BCPP RNA-seq cohort using the RTN (Reconstruction of Transcriptional regulatory Networks and analysis of regulons) package (version 2.14.1) **(20)**. Briefly, the normalised gene expression along with sample phenotype traits (which included tumour grade, stage, consensus cluster membership, tumour mutational burden, copy number burden etc.) are evaluated for inferring transcriptional network activity for the regulon list provided. By computing mutual information content for each of the regulon to be tested and its target genes, using permutation analysis to filter out non-significant associations and finally using the ARACNe algorithm to further select direct as opposed to indirect interactions, regulon activities are inferred that are specific to the input gene expression. The direction of the activity (positive or negative) is inferred then through applying a two-tailed GSEA test that returns the regulons with statistically significant activity. For the regulons with statistical significance, a per-sample activity score was estimated to correlate with sample phenotypes such as tumour grade and stage.

**Estimation of immune cell composition**

Immune cell composition of the bulk tumour tissue was estimated by deconvolution of the RNA-sequencing read counts using ConsensusTME (version 0.0.1.9) **(21)**. Briefly, rather than using a single method or gene set, ConsensusTME is a consensus of immune cell deconvolution estimates from multiple methods like CIBERSORT, ESTIMATE, MCP-Counter, xCell etc. Using enrichment scores calculated using single-sample GSEA (ssGSEA) for each of the gene sets across the methods, a normalised enrichment score (NES) is calculated for 18 different immune cell types. The NES thus obtained from ConsensusTME is then taken as a sample phenotype measure while correlating with gene expression or DNA alteration level subtypes.

**Circular RNA prediction**

Prediction of circular RNA (circRNA) candidates inferred from back-splicing events in RNA-sequencing were carried out as detailed previously **(22)**. Briefly, the DCC algorithm **(23)** was used and circRNA candidates with read-depth support >=4 were retained. The ratio of the circRNA to the linear RNA (host gene) were calculated and Analysis of Variance (ANOVA) was applied (within R) to identify those with significant difference across the RNA consensus clusters (at adjusted p-value 0.05).

**Comparison of BCPP expression classes with UROMOL 2021**

The UROMOL 2021 NMIBC classification assignment for the BCPP RNA-sequencing samples was obtained from the website (http://134.157.229.105:3838/BLCAclassify/). Correspondence between the two classification systems was assessed using molecular aberration indices of TMB (tumour mutation burden), CNB (copy number burden), estimates of ploidy, tumour cellularity, HRD (homologous recombination mediated DNA repair deficiency) score and tumour cell infiltration (immune score). Boxplots were made for visualisation and non-parametric Kruskal-Wallis test performed in R (ver. 4.0.2)

**Survival analysis**

We investigated the effects of tumour grade, stage, patient age, gender, summary molecular aberration indices (tumour mutational burden, copy number burden, APOBEC mutational activity and homologous recombination repair deficiency) and mutational status of specific genes, against the progression-free survival (PFS; progression to MIBC). R packages survival (version 3.2.3) and survminer (version 0.4.8) were used to implement the Cox Proportional Hazards model was used to perform multivariate survival analysis using the coxph function and forest plots were created using ggforest function (tidyverse version 1.3.0).

**References**

1. Martin, M., Cutadapt removes adapter sequences from high-throughput sequencing reads. 2011, 2011. 17(1): p. 3.
2. Bolger, A.M., M. Lohse, and B. Usadel, Trimmomatic: a flexible trimmer for Illumina sequence data. Bioinformatics, 2014. 30(15): p. 2114-2120.
3. Li, H. and R. Durbin, Fast and accurate short read alignment with Burrows–Wheeler transform. Bioinformatics, 2009. 25(14): p. 1754-1760.
4. Van der Auwera, G.A., et al., From FastQ data to high confidence variant calls: the Genome Analysis Toolkit best practices pipeline. Curr Protoc Bioinformatics, 2013. 43(1110): p. 11.10.1-11.10.33.
5. Karczewski, K.J., et al., The mutational constraint spectrum quantified from variation in 141,456 humans. Nature, 2020. 581(7809): p. 434-443.
6. McLaren, W., et al., The Ensembl Variant Effect Predictor. Genome Biology, 2016. 17(1): 122.
7. Rosenthal, R., et al., DeconstructSigs: delineating mutational processes in single tumors distinguishes DNA repair deficiencies and patterns of carcinoma evolution. Genome Biol, 2016. 17: p. 31.
8. Talevich, E., et al., CNVkit: Genome-Wide Copy Number Detection and Visualization from Targeted DNA Sequencing. PLoS Comput Biol, 2016. 12(4): p. e1004873.
9. Mermel, C.H., et al., GISTIC2.0 facilitates sensitive and confident localization of the targets of focal somatic copy-number alteration in human cancers. Genome Biology, 2011. 12(4): p. R41.
10. Mayakonda, A., et al., Maftools: efficient and comprehensive analysis of somatic variants in cancer. Genome Res, 2018. 28(11): p. 1747-1756.
11. Favero, F., et al., Sequenza: allele-specific copy number and mutation profiles from tumor sequencing data. Ann Oncol, 2015. 26(1): p. 64-70.
12. Sztupinszki, Z., et al., Migrating the SNP array-based homologous recombination deficiency measures to next generation sequencing data of breast cancer. npj Breast Cancer, 2018. 4(1): p. 16.
13. Dobin, A., et al., STAR: ultrafast universal RNA-seq aligner. Bioinformatics, 2013. 29(1): p. 15-21.
14. Law, C. W., et al., Voom: precision weights unlock linear model analysis tools for RNA-seq read counts. Genome Biology, 2014. 15(2): R29.
15. Kuleshov, M.V., et al., Enrichr: a comprehensive gene set enrichment analysis web server 2016 update. Nucleic Acids Res, 2016. 44(W1): p. W90-7.
16. Luo, W., et al., GAGE: generally applicable gene set enrichment for pathway analysis. BMC Bioinformatics, 2009. 10(1): p. 161.
17. Korotkevich, Gennady, et al. "Fast gene set enrichment analysis." BioRxiv (2021): 060012.
18. Wilkerson, M.D. and D.N. Hayes, ConsensusClusterPlus: a class discovery tool with confidence assessments and item tracking. Bioinformatics, 2010. 26(12): p. 1572-3.
19. Robertson, A.G., et al., Comprehensive Molecular Characterization of Muscle-Invasive Bladder Cancer. Cell, 2017. 171(3): p. 540-556.e25.
20. Castro, M.A., et al., RTN: Reconstruction of transcriptional networks and analysis of master regulators (R/Bioconductor package), 2016b.
21. Jiménez-Sánchez, A., O. Cast, and M.L. Miller, Comprehensive Benchmarking and Integration of Tumor Microenvironment Cell Estimation Methods. Cancer Research, 2019. 79(24): p. 6238-6246.
22. Goel, A., et al., Back-Splicing Transcript Isoforms (Circular RNAs) Affect Biologically Relevant Pathways and Offer an Additional Layer of Information to Stratify NMIBC Patients. Frontiers in Oncology, 2020. 10(812).
23. Cheng, J., F. Metge, and C. Dieterich, Specific identification and quantification of circular RNAs from sequencing data. Bioinformatics, 2015. 32(7): p. 1094-1096.
